# A Series of Lanthanide Coordination Polymers as Luminescent Sensors for Selective Detection of Inorganic Ions and Nitrobenzene

**DOI:** 10.3390/molecules29143438

**Published:** 2024-07-22

**Authors:** Miao Wu, Juan Song, Yun-Long Zhou, Hui-Hui Chen, Bo-Feng Duan, Ling-Xia Jin, Chuan-Qing Ren, Jiu-Fu Lu

**Affiliations:** 1 Shaanxi Key Laboratory of Catalysis, College of Chemical & Environment Science, Shaanxi University of Technology, Hanzhong 723001, China; wumiao06242022@163.com (M.W.); zhouyunlong@snut.edu.cn (Y.-L.Z.); chenhuihui@snut.edu.cn (H.-H.C.); jinlx@snut.edu.cn (L.-X.J.); renchuanqing@snut.edu.cn (C.-Q.R.); jiufulu@snut.edu.cn (J.-F.L.); 2 Trine Engineering Institute, Shaanxi University of Technology, Hanzhong 723001, China; duanbofeng@snut.edu.cn

**Keywords:** lanthanide coordination polymers, Co^2+^ ion, Cu^2+^ ion, nitrobenzene, fluorescence sensor

## Abstract

Seven new lanthanide coordination polymers, namely [Ln(cpt)_3_H_2_O)]n(Ln = La (**1**), Pr (**2**), Sm (**3**), Eu (**4**), Gd (**5**), Dy (**6**), and Er (**7**)), which were synthesized under hydrothermal conditions using 4′-(4-(4-carboxyphenyloxy)phenyl)-4,2′:6′,4′-tripyridine (Hcpt) as the ligand. The crystal structures of these seven complexes were determined using single-crystal X-ray diffraction, and they were found to be isostructural, crystallizing in the triclinic P$\overset{-}{1}$ space group. The Ln(III) ions were nine-coordinated with tricapped trigonal prism coordination geometry. The Ln(III) cations were coordinated by carboxylic and pyridine groups from (cpt)^−^ ligands, forming one-dimensional ring-chain structures. Furthermore, the luminescent properties of complexes **1**–**7** were investigated using fluorescent spectra in the solid state. The fluorescence sensing experiments demonstrated that complex **4** exhibits high selectivity and sensitivity for detecting Co^2+^, Cu^2+^ ions, and nitrobenzene. Moreover, complex **3** shows good capability for detecting Cu^2+^ ions and nitrobenzene. Additionally, the sensing mechanism was also thoroughly examined through theoretical calculations.

## 1. Introduction

With the continuous development of industry and human social activities, a significant amount of heavy metal ions and toxic small molecules are being discharged into our living environment, leading to serious environmental and public health issues [1,2,3,4,5]. The sensing and detection of metal ions play a crucial role in environmental and ecological systems, with direct implications for human health and daily life. In biological systems, the Cu^2+^ ion is essential, and its imbalance may be associated with metabolic disorders such as Parkinson’s, Wilson’s, and Alzheimer’s [6,7,8,9,10,11]. On the other hand, Co^2+^ has toxic effects and can lead to serious health problems including cardiomyopathy, hypothyroidism, peripheral neuropathy, and respiratory disorders [12,13,14]. Meanwhile, nitrobenzene (NB), a toxic industrial chemical used as a precursor for explosives, pesticides, and synthetic rubbers and in the production of dyes and pharmaceuticals, poses significant safety risks [15]. Furthermore, NB can be easily inhaled, ingested, and absorbed through the skin, leading to methemoglobinemia, hemolytic anemia, splenic congestion, and disorders of the central nervous system (CNS) [16,17,18]. Therefore, researchers are dedicated to exploring simple and practical methods for detecting low concentrations of Co^2+^, Cu^2+^, and NB. However, the majority of testing methods require costly instruments and skilled technicians. Therefore, it remains crucial for the protection of the environment and human health to develop efficient methods and design multifunctional materials with high sensitivity and selectivity for detecting Co^2+^, Cu^2+^, and NB.

In recent years, there has been significant attention on the design and construction of lanthanide coordination polymers (Ln-Cps) as luminescent sensors due to their intriguing architectures, high sensitivity, excellent selectivity, rapid response time, and cost effectiveness [19,20,21]. The Ln-Cps have been widely reported as luminescent sensors for the detection of anions [22,23,24,25], cations [26], biological molecules [27] and organic molecules [28,29]. The 4f orbitals in lanthanide ions are shielded by the filled 5p^6^6s^2^ subshells, leading to unique optical properties. Lanthanide coordination polymers exhibit high color purity, narrow band emission, long lifetime, and high quantum yields [30]. However, the absorption coefficients and luminescence intensities of pure rare-earth ions are significantly low, and the 4f–4f transitions of lanthanide are Laporte-forbidden. To mitigate these limitations, organic ligands with suitable chromophores can be selected to absorb Uv–vis light and transfer excitation energy from the ligands to lanthanide ions through an “antenna effect”, thereby shielding Ln(III) ions from vibrational coupling that dampens luminescence [31]. The selection of a suitable ligand is crucial for the construction of lanthanide coordination polymers skeletons, as it determines the structure and chemical environment of the resulting complexes. Choosing long and flexible ligands can contribute to the production of interesting topologies and rich properties. After considering all the aforementioned factors, we chose 4′-(4-(4-carboxyphenoxy)phenyl)-4,2′:6′,4″-tripyridine as the ligand based on the following considerations: (1) The Hcpt ligand contains abundant coordination sites, including the nitrogen atom in pyridine and the oxygen atoms of carboxylic acid, all of which can form strong bonds with lanthanide ions and exhibit variable coordination patterns in self-assembly. (2) In addition to the tripyridine and carboxylic group, the Hcpt ligand also contains a phenoxy group, which increases the length and rigidity of the ligand. (3) The Hcpt ligand contains a large π-conjugated system, enabling efficient energy transfer to the lanthanide center for indirect excitation and light emission during relaxation. Additionally, it enhances the thermal stability of lanthanide complexes. (4) To the best of our knowledge, the coordination chemistry and structural properties based on the Hcpt ligand have not been previously reported.

In order to construct novel lanthanide coordination polymers with unique structures and high sensitivity and selectivity for detecting Co^2+^, Cu^2+^, and NB, we employed 4′-(4-(4-carboxyphenyloxy) phenyl)-4,2′:6′,4′-tripyridine (Hcpt) as a ligand to undergo hydrothermal reaction with lanthanide ions, resulting in the isolation of seven new lanthanide coordination polymers: [Ln(cpt)_3_H_2_O)]n(Ln = La (**1**), Pr (**2**), Sm (**3**), Eu (**4**), Gd (**5**), Dy (**6**), and Er (**7**)). We herein present the synthesis of these compounds, their crystal structures, as well as their potential applications as fluorescence sensing materials and the underlying sensing mechanism.

## 2. Results and Discussion

### 2.1. Structural Description of Complexes ***1***–***7***

**Figure 1:**
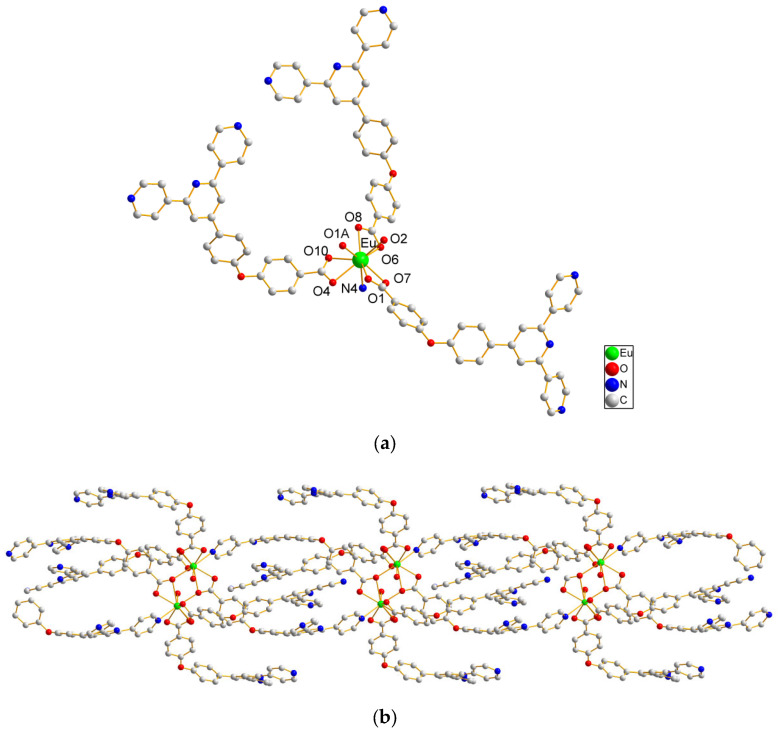

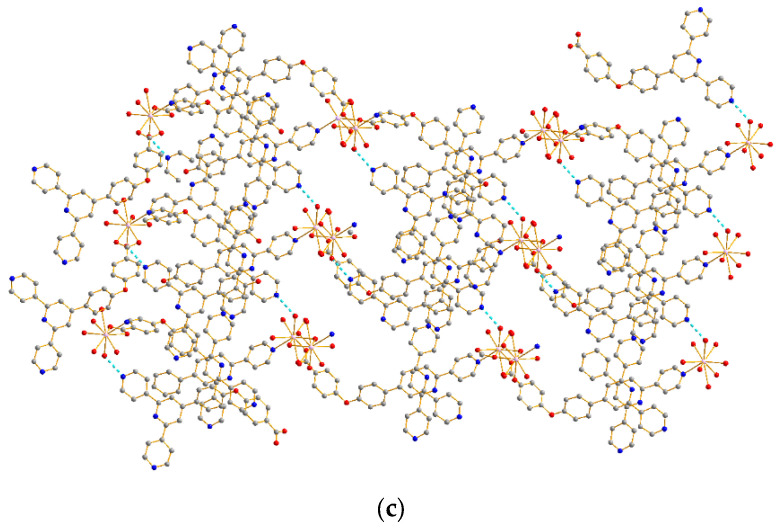
(**a**) Coordination environment of the Eu^III^ cations in complex **4**. (**b**) The one-dimensional ring-chain structure of complex **4**. (**c**) Two-dimensional supramolecular structure of complex **4**.

The structures of complexes **1**–**7** are isostructural; therefore, only the detailed description of complex **4** is provided. The single-crystal X-ray analysis revealed that complex **4** exhibits a one-dimensional ring-chain structure. The asymmetric unit of **4** contains one Eu(III) cation, four (cpt)^−^ ligands, and a water molecule. The coordination polyhedron around Eu(III) is a nine-coordinated dodecahedron consisting of seven oxygen (O_1_, O_1_A, O_7_, O_6_, O_8_, O_4_, and O_10_) atoms and one nitrogen (N_4_) from four (cpt)^−^ ligands as well as one oxygen (O_2_) from one coordinated water molecule (Figure 1a). The Eu–O bond lengths fall within the range of 2.4090(19) Å–2.725(2) Å, while the Eu–N bond lengths are 2.715(2) Å, demonstrating strong consistency with previous research findings [32].

**Scheme 1:**
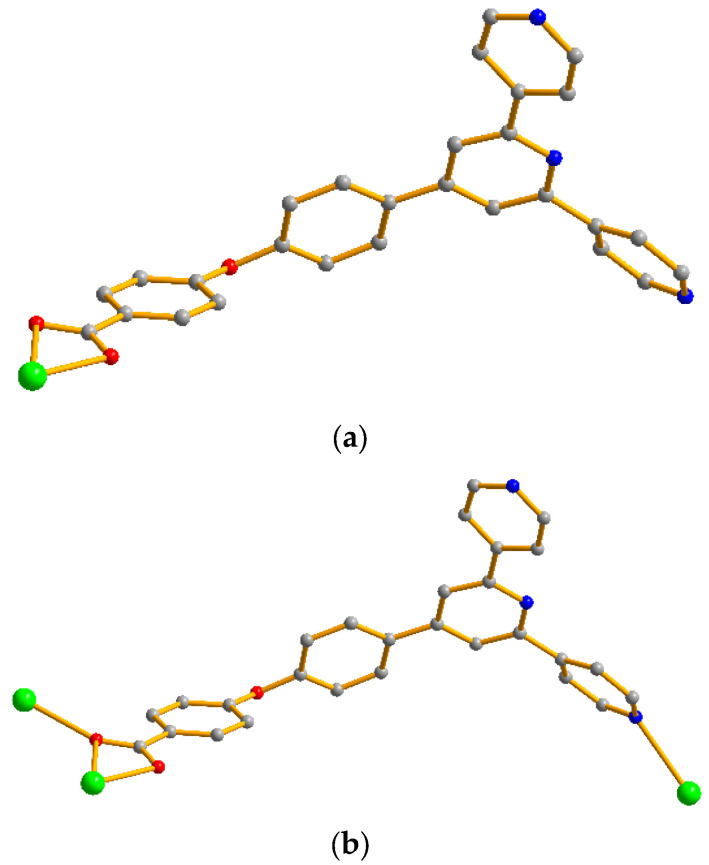
Coordination modes of the (cpt)^−^ ligand. (**a**) a bidentate coordination fashion (**b**) a tridentate coordination fashion.

It is worth noting that the (cpt)^−^ anion adopts two different coordination fashions: (a) a bidentate coordination fashion in which the carboxylic group acts as a bidentate chelating coordination mode, and the terpyridyl moiety is free (Scheme 1a), and (b) a tridentate coordination fashion in which the carboxylic acid adopts *μ*_2_–*η*^1^:*η*^2^ coordination mode, and the terpyridyl moiety acts as the monodentate-chelating coordination mode, coordinating to another atom Eu(III) (Scheme 1b). The Eu(III) atoms are linked by (cpt)^−^ ligands in the two coordination modes, resulting in the formation of a 1D infinite ring-chain structure (Figure 1b). Furthermore, these adjacent chains are further connected via hydrogen bonding interactions between coordinated O atoms and N atoms of (cpt)^−^ ligand (O_2_–H_2_B…N_2_, 2.728 Å), resulting in the formation of a 2D supramolecular structure (Figure 1c). The crystallographic refinement parameters are provided in Tables S1 and S2.

### 2.2. Luminescent Properties

**Figure 2:**
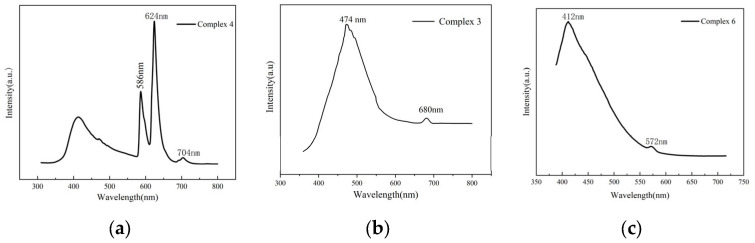
(**a**) Emission spectra of complex **4**. (**b**) Emission spectra of complex **3**. (**c**) Emission spectra of complex **6**.

The solid-state luminescent behaviors of the free ligand and complexes **1**–**7** were analyzed at ambient temperature when excited at 390 nm. The fluorescence emission of the free ligand was at 474 nm (Figure S1a). For complex **4**, the three sharp characteristic peaks shown in Figure 2a belong to the transitions of ^5^D_0_→^7^F_1_ (586 nm), ^5^D_0_→^7^F_2_ (624 nm), and ^5^D_0_→^7^F_4_ (704 nm). The intensity of the ^5^D_0_→^7^F_2_ transition (electric dipole) was stronger than that of ^5^D_0_→^7^F_1_ transition (magnetic dipole), indicating an asymmetric coordination environment for the Eu^3+^ ion, as confirmed by crystallographic analyses. For complex **3**, only one characteristic emission band of the Sm(III) cation could be observed in the emission spectra (Figure 2b), which was attributed to ^4^G_5/2_→^6^H_9/2_ (680 nm). For complex **6**, the emission spectra (Figure 2c) revealed the presence of one weak characteristic emission band of the Dy(III) cation, which could be attributed to ^4^F_9/2_→^6^H_13/2_ (572 nm) transitions. Compared with the emission spectra of the three complexes, the energy transfers from the ligand to Eu(III) were more efficient than those to Sm(III) and Dy(III). The complexes **1**, **2**, **5**, and **7** displayed the characteristic emission peaks of the ligand at 409 nm (**1**), 450 nm (**2**), 456 nm (**5**), and 436 nm (**7**), all exhibiting a blue shift compared to the free ligand (Figure S1b–e).

**Figure 3:**
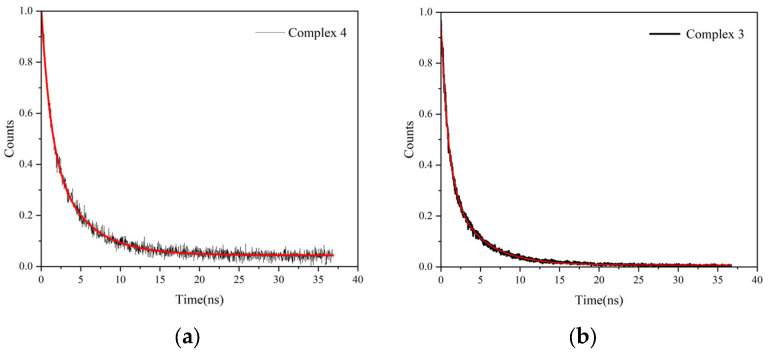
(**a**) Luminescence decay curves of complex **4**. (**b**) Luminescence decay curves of complex **3**.

**Figure 4:**
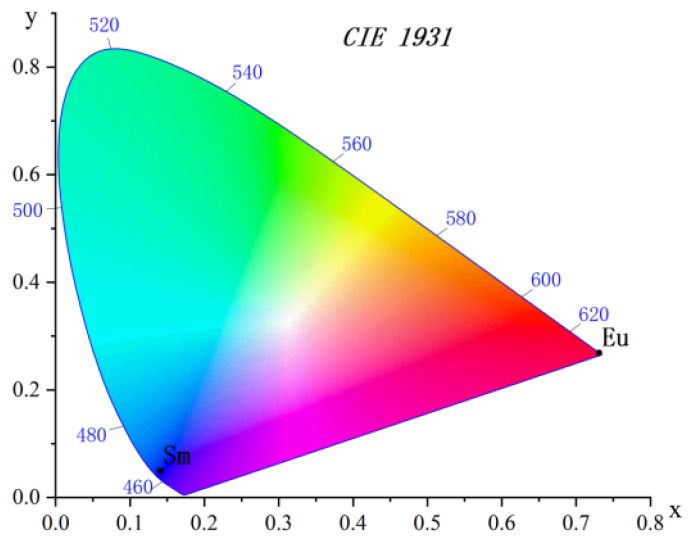
CIE chromaticity diagrams for complex **4** and **3**.

Furthermore, the fluorescence lifetime of coordination polymers **4** and **3** were monitored at 624 nm and 680 nm, respectively. The fluorescence decays of the coordination polymers **4** and **3** in the solid state at ambient temperature were fitted into double-exponential decay laws with the following formula: I = A_1_ exp(t/τ_1_)+ A_2_ exp(t/τ_2_), where τ_1_ and τ_2_ are defined as the fast and slow components of the luminescence lifetimes, while A_1_ and A_2_ denote the preexponential factors (Figure 3). The fitted fluorescence lifetimes τ_1_ and τ_2_ were 1.15 ns (21.98%) and 4.52 ns (78.02%) for **4** and 1.05 ns (34.10 %) and 4.60 ns (65.90%) for **3**. As a result, the average decay times (τ*) was determined by the following equation: τ* = (A_1_τ_1_^2^ + A_2_τ_2_^2^)/(A_1_τ_1_ + A_2_τ_2_), giving the corresponding average lifetimes of 3.78 ns and 3.39 ns. The CIE chromaticity coordinates of the corresponding samples were (0.7309, 0.2691) for complex **4** and (0.1400, 0.0495) for complex **3**, as illustrated in Figure 4.

### 2.3. Detection of Metal Ions

**Figure 5:**
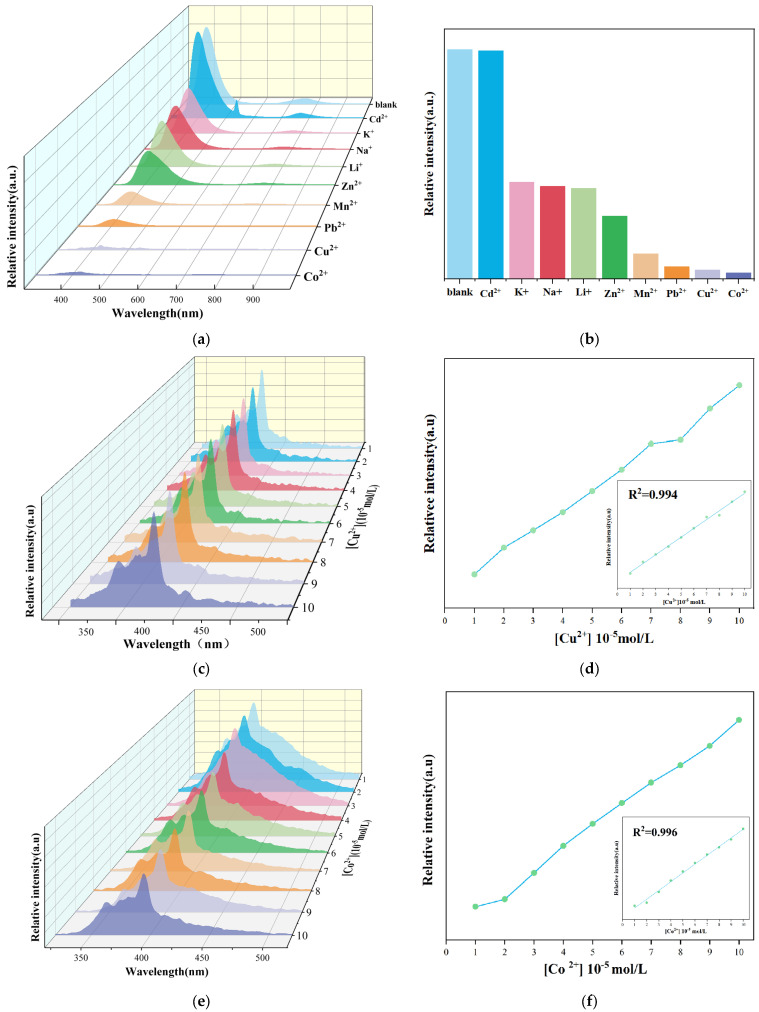
(**a**) Emission spectra of complex **4** in diverse metal ions. (**b**) Comparisons for intensities of complex **4** in different metal ions. (**c**,**e**) Luminescence quenching of complex **4** dispersed in DMF with gradual addition of Cu^2+^ and Co^2+^ ions, respectively. (**d**,**f**) The fitting of the fluorescence change value of complex **4** in Cu^2+^ and Co^2+^ ions, respectively.

**Figure 6:**
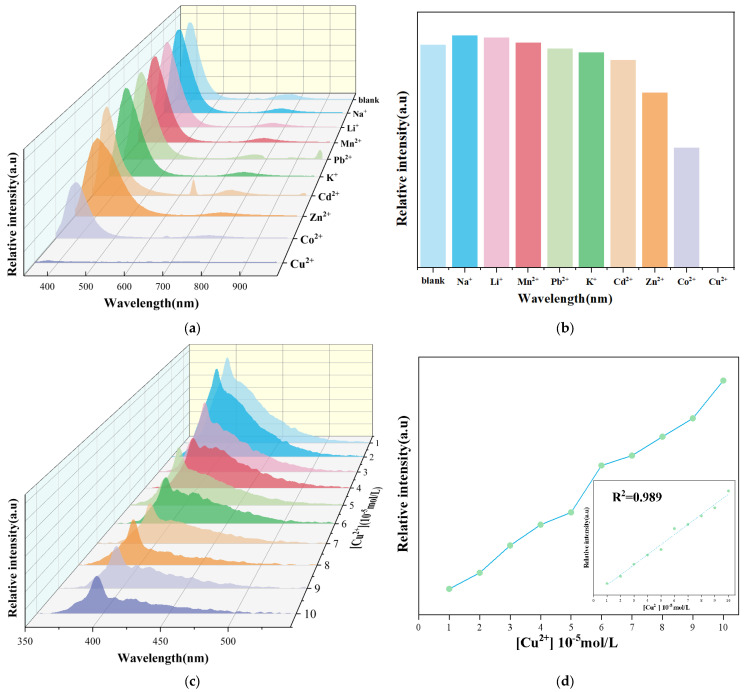
(**a**) Emission spectra of complex **3** in diverse metal ions. (**b**) Comparisons for intensities of complex **3** in different metal ions. (**c**) Luminescence quenching of complex **3** dispersed in DMF with gradual addition of Cu^2+^ ion. (**d**) The fitting of the fluorescence change value of complex **3** in Cu^2+^ ion.

The structures of complexes **1**–**7** are isostructural, and they show a gradual decrease in ionic radius with increasing atomic number (also known as the lanthanide contraction); the resulting applications determine to a large extent these lanthanide ions themselves [33,34]. Studies on luminescent lanthanides have concentrated on Sm(III), Eu(III), Tb(III), and Dy(III) ions in the visible region [35], and due to their relatively excellent fluorescence properties, complexes **4** and **3** were selected for investigating their selective sensing behavior towards metal ions. Finely ground samples of **4**/**3** were suspended in equal volumes of different DMF solutions, each containing a concentration of 1.0 × 10^−2^ mol/L of specific M(NO_3_)n species (M = Li^+^, Pb^2+^, Mn^2+^, Co^2+^, K^+^, Na^+^, Cu^2+^, Cd^2^+, and Zn^2+^), for luminescence studies at ambient temperature. As shown in Figure 5a,b, the fluorescence emission intensity of complex **4** was most significantly affected by Cu^2+^ and Co^2+^ ions, showing a more pronounced decrease compared to other ions. The distinct sensing behavior on Cu^2+^ and Co^2+^ from other cations showed that complex **4** may be employed as an efficient sensor to distinguish Cu^2+^ and Co^2+^ ions. Interestingly, the luminescence intensity of **3** was quenched close to 0 by only Cu^2+^ ions (Figure 6a,b), suggesting that complex **3** can effectively detect Cu^2+^ through the phenomenon of fluorescence quenching.

In order to further evaluate the sensitivity of complex 4 in sensing Cu^2+^ and Co^2+^ as well as the sensing sensitivity of **3** towards Cu^2+^, concentration gradient experiments were conducted with Cu^2+^ and Co^2+^ concentrations ranging from 10 to 100 μM. The emission intensity of **4** and **3** gradually decreased as the concentration of Co^2+^/Cu^2+^ and Cu^2+^ increased, as demonstrated in Figure 5c,e and Figure 6c. The fluorescence intensity of complex **4** shows a good linear relationship with ion concentration (R^2^ = 0.994 for Cu^2+^ and 0.996 for Co^2+^) in Figure 5d,f. Additionally, the fluorescence intensity of complex **3** demonstrates a good linear relationship with the concentration of Cu^2+^ (R^2^ = 0.989) in Figure 6d. Furthermore, the quenching efficiency (K_sv_) can be quantitatively calculated using the Stern–Volmer equation: I_0_/I = 1 + K_sv_[M], where I_0_ and I represent the emission intensities of the reference sample and complexes, respectively, and [M] represents the concentration [36,37]. The K_sv_ values for complex **4** towards Cu^2+^ and Co^2+^ were calculated to be 4.9 × 10^4^ M^−1^ and 9.4 × 10^4^ M^−1^, respectively, while the Ksv values for complex **3** towards Cu^2+^ were calculated to be 3.53 × 10^4^ M^−1^. The limit of detection (LOD) was further calculated according to 3σ/k (σ is the standard deviation of 10 blank tests; k is the slope of the linear relationship), which were 8.13 × 10^−5^ mol/L for Cu^2+^, 4.4 × 10^−5^ mol/L for Co^2+^, and 1.13 × 10^−5^ mol/L for Cu^2+^. These values are consistent with those reported for some complex sensors designed to detect Co^2+^ and Cu^2+^ [38,39].

### 2.4. Sensing of Organic Small Molecules

**Figure 7:**
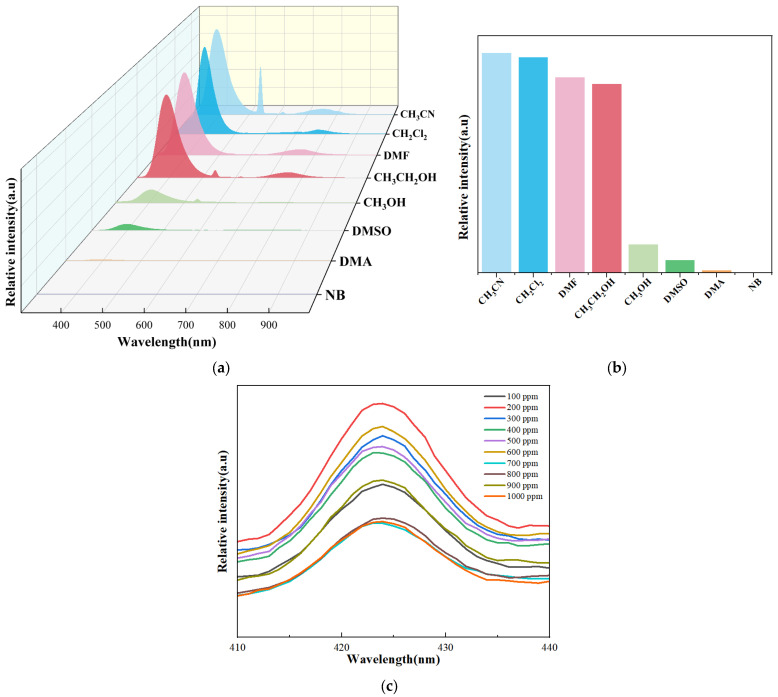
(**a**) Emission spectra of complex **4** in diverse analytes. (**b**) Comparisons for intensities of **4** in diverse analytes. (**c**) Emission spectra of complex **4** in CH_3_CN with different concentrations of NB.

**Figure 8:**
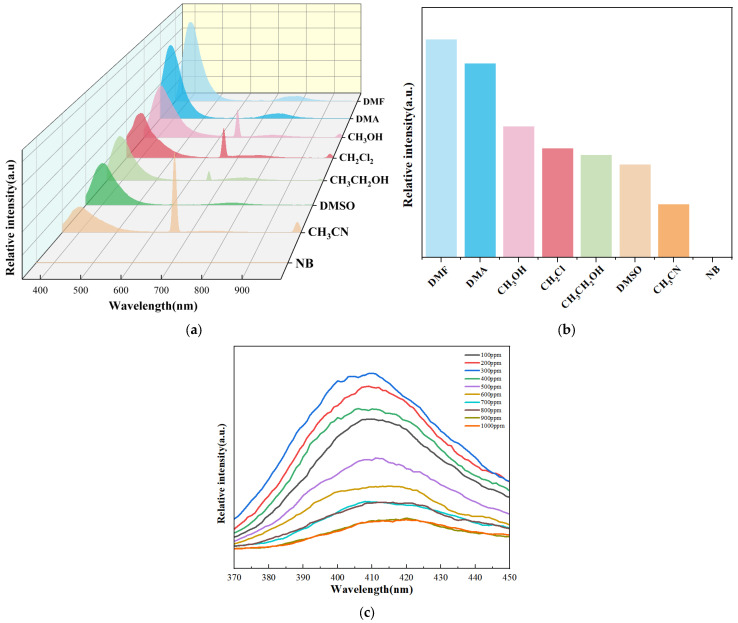
(**a**) Emission spectra of complex **3** in diverse analytes. (**b**) Comparisons for intensities of **3** in diverse analytes. (**c**) Emission spectra of complex **3** in DMF with different concentrations of NB.

The rapid and sensitive detection of toxic small molecules is crucial due to their high toxicity, which poses a potential threat to environmental safety, public health, and human security. After being inspired by this concept, we evaluated the sensing behaviors of complexes **4** and **3** towards various organic small molecules using fluorescence, with the addition of different organic solvents such as CH_3_CN, NB, CH_3_CH_2_OH, CH_3_OH, DMA, DMF, DMSO, and CH_2_Cl_2_ to the suspensions of **4/3**. As illustrated in Figure 7a,b and Figure 8a,b, different degrees of fluorescence intensity quenching were observed for **4** and **3** upon the addition of all selected analytes. Among them, only NB thoroughly quenched the emission of complexes **4** and **3**, with a quenching efficiency of approximately 99%. The luminescence of coordination polymers **4** and **3** was significantly influenced by the presence of solvent molecules. Specifically, the CH_3_CN suspension of **4** exhibited the strongest emission band, while the DMF suspension of **3** exhibited the strongest emission band at ambient temperature. Thus, fluorescent detection experiments were performed in CH_3_CN solution of complex **4** and DMF solution of complex **3**. To further investigate the sensitivity of fluorescence quenching characteristics for NB, the emissive response was recorded by gradually increasing the concentration of NB in the suspension of coordination polymer **4** dispersed in CH_3_CN and **3** dispersed in DMF. As shown in Figure 7c and Figure 8c, the fluorescence bursting phenomenon of complex **4** was most pronounced at a concentration of 700 ppm NB, with a fluorescence bursting efficiency reaching 99.99%. For complex **3**, at a concentration of 1000 ppm NB, the quench efficiency reached as high as 99.99%. Although there is no strict systematic trend in the Figure 7c and Figure 8c, the general trend is still that the quenching effect increases with the increase of concentration, indicating that **4** and **3** are sensitive in detecting trace quantities of NB.

### 2.5. PXRD and FT-IR

To confirm the purities of the bulk samples, PXRD experiments were carried out for lanthanide coordination polymers **1**–**7** (Figure S2). By comparing the PXRD experimental spectrum of the complex with the theoretical spectrum obtained from fitting single-crystal data, it was observed that the diffraction peak positions in both spectra were essentially consistent. Additionally, no extra diffraction peaks were observed in the experimental spectrum, confirming the purity of the complex obtained. The difference in diffraction peak intensity between the experimental and theoretical spectra can be attributed to the uneven orientation distribution of the powder sample during the diffraction experiment. Moreover, PXRD patterns of complexes **4** and **3** after sensing tests were measured, which are in good agreement with the synthesized **4** and **3**, suggesting that the host skeleton remains unchanged after immersion in aqueous solution containing different metal ions (Figure S3). Besides that, the FT-IR spectra of the recycled complexes **4** and **3** were also recorded. And these infrared spectra after processing were highly matched to those before treatment (Figure S4). This indicates that the structure of the complexes **4** and **3** did not collapse during fluorescence detection, and the detected complexes can be recycled for subsequent experiments.

### 2.6. Theoretical Studies

**Figure 9:**
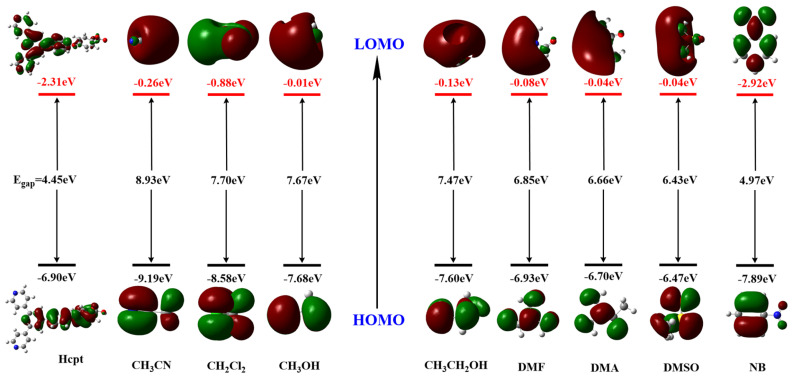
HOMO and LUMO energy levels of ligand and the selected analytes.

In order to explain the high selectivity and sensitivity of complexes **4** and **3** towards NB, an investigation into the mechanism of fluorescence quenching was conducted. It is well known that fluorescence quenching occurs through mechanisms such as photo-induced electron transfer, long-range energy transfer processes, dynamic-static quenching mechanisms, or a combination of them. The lack of porosity in complexes **4** and **3** excludes the mechanisms of guest-induced fluorescence quenching. The observed fluorescence quenching phenomenon can be attributed to the photoinduced electron transfer from the excited state of the two compounds to the electron-deficient NB, which belongs to the surface adsorption. The coordination polymers can be considered as giant “molecules”, and their valence-band (VB) and conduction-band (CB) energy levels can be described in a fashion similar to that used for molecular orbitals (MOs) [40,41,42,43,44,45,46,47,48,49,50,51,52,53,54,55,56,57,58,59,60,61,62]. The low-lying π* orbitals of nitrobenzene, which are the lowest unoccupied MOs (LUMOs), are stabilized by the -NO_2_ substituent. Their orbital energies always fall below the conduction band (CB) of coordination polymers. This leads to the driving force for electron transfer from the coordination polymers to nitrobenzene, thus resulting in fluorescence quenching. Figure 9 exhibits the highest occupied molecular orbital (HOMO) and lowest unoccupied molecular orbital (LUMO) energy levels of ligand and the selected analytes, including NB, as determined through density functional theory (DFT) calculations using the B3LYP/6-31 + G(d) level of theory with the Gaussian 03 package. The LUMO energy of the electron-rich ligand was calculated to be −2.31 eV, which is higher than that of NB (−2.92 eV) and lower than those of other tested analytes, indicating that there are excited-state electrons transferred from electron-rich coordination polymers to electron-deficient NB [63,64].

The fluorescence of complex **4** and complex **3** results from the antenna effect, in which the Hcpt ligand adsorbs energy through vibronic coupling with Ln^3+^ and subsequently transfers this energy to Ln^3+^, resulting in the fluorescence of Ln^3+^.The quenching efficiency of NB may be attributed to the competition for absorption of the light source energy between NB and the Hcpt ligand. NB acts as a filter for the light adsorbed by the Hcpt ligand, thus decreasing the probability of energy transfer from Hcpt to Ln^3+^, consequently leading to the quenching of the fluorescence of the complexes. Therefore, compared to other organic solvents, complexes **4** and **3** exhibit much higher fluorescence quenching responses towards NB due to favorable electron and energy transfer mechanisms.

### 2.7. Hirshfeld Surface Analysis

**Figure 10:**
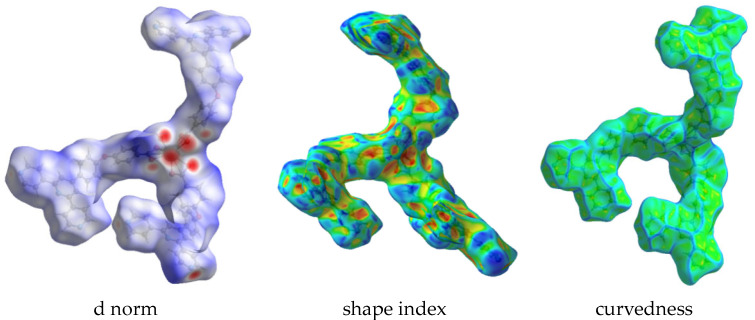
Hirshfeld surface mapped with dnorm (**left**), shape index (**middle**), and curvedness (**right**) for complex **4**.

**Figure 11:**
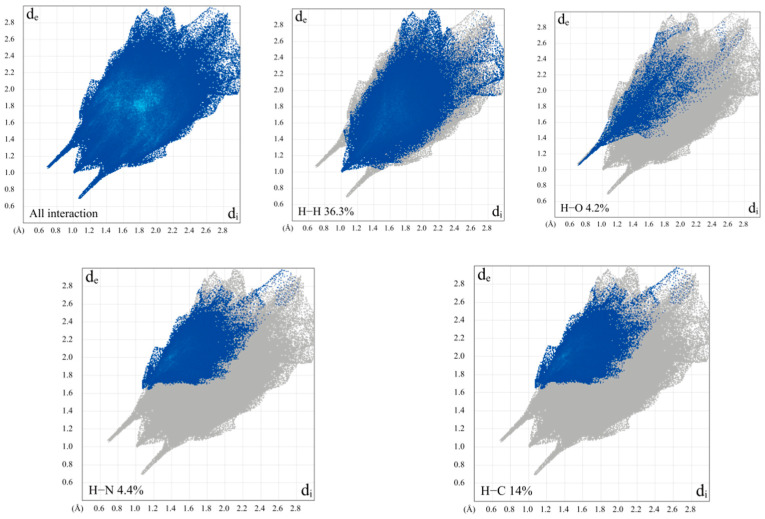
Two-dimensional (2D) fingerprint plots of Hirshfeld surfaces for complex **4**.

To analyze the various interactions corresponding to the crystal structures, the Hirshfeld surface and 2D fingerprints plot of the complexes **1**–**7** were calculated based on the crystallographic information file (CIF) using the CrystalExplorer program (Figure 10 and Figure S5). The Hirshfeld surface analysis of complex **4** is described in detail due to the isostructural nature of complexes **1**–**7**. As illustrated in Figure 10, the colors red, white, and blue correspond to the relationship between forces present at that location and the sum of the van der Waals radii [65]. The 2D fingerprints of the complexes provide a clearer visualization of the interactions between corresponding atoms (Figure 11). The H…H inter-atomic contacts make the most significant contribution to the crystal packing of complex 4 (36.3%). This finding can be attributed to the predominance of hydrogen atoms in the crystal. The H…C interaction located in the second ladder contributes to 14% of the complex, while the H…O exposure accounts for 4.2%. In addition to these interaction forces, the H…N interactions also contribute 4.4% of the total Hirshfeld surface area among all interactions, attributed to the presence of coordinated nitrogen atoms in the complex. In comparison, the H…H interaction contributes the most to the structures of the complex. In summary, four forces significantly contributed to the construction and stabilization of molecular structures [66].

## 3. Materials and Methods

### 3.1. Materials and General Method

All chemicals and reagents were purchased commercially and used without further purification. Infrared spectra were recorded on a Bruker EQUINOX55 spectrometer (Billerica, MA, USA) as KBr pellets in the range of 4000–500 cm^−1^. Fluorescence spectra were performed on a Hitachi F-4500 fluorescence spectrophotometer (Tokyo, Japan) at room temperature. The crystal structure was measured using a BRUKER SMART APEX-IICCD X-ray single-crystal diffractometer. Powder X-ray diffraction (PXRD) patterns were recorded on a Bruker D8 Advance instrument with Cu Kα radiation (λ = 0.71073 Å) in the range of 2θ = 5–50° at room temperature.

### 3.2. Synthesis of Complexes

Synthesis of [La(cpt)_3_(H_2_O)] (**1**).

A mixture of the ligand Hcpt (22.2 mg, 0.05 mmol) and La(NO_3_)_3_·6H_2_O (43.30 mg, 0.10 mmol) was prepared by dissolving in 10 mL of distilled water; the mixture was stirred at room temperature for 30 min before being transferred into a 25 mL Teflon-lined stainless steel autoclave and heated to 180 °C for 72 h. The reaction system was gradually cooled to ambient temperature at a rate of 5 °C/h, resulting in the formation of colorless, block-shaped crystals of complex 1 in ca. 67.33% based on La. Elemental analysis (%) calculated forC_84_H_56_LaN_9_O_10_ (M = 1490.28): C = 67.70, H = 3.79, N = 8.46; Found: C = 66.58; H = 3.56; N = 8.04. IR (KBr pellets, cm^−1^): 3320, 1698, 1602, 1513, 1395, 1237, 867, 824, 631 (Figure S6a).

Synthesis of [Pr(cpt)_3_(H_2_O)] (**2**).

Complex **2** was obtained by a similar procedure to that for **1**, except that Pr(NO_3_)_3_·6H_2_O (43.50 mg, 0.1 mmol) was used instead of La(NO_3_)_3_·6H_2_O, yielding colorless, block-shaped crystals of complex 2 in ca. 68.51% based on Pr. Elemental analysis (%) calculated for C_84_H_56_PrN_9_O_10_ (M = 1492.285): C = 67.61, H = 3.78, N = 8.45; Found: C = 66.72, H = 3.56, N = 7.84. IR (KBr pellets, cm^−1^): 3415, 1596, 1498, 1395, 1238, 1006, 876, 824, 631 (Figure S6b).

Synthesis of [Sm(cpt)_3_(H_2_O)] (**3**).

Complex **3** was obtained by a similar procedure to that for **1**, except that Sm(NO_3_)_3_·6H_2_O (22.22 mg, 0.05 mmol) was used instead of La(NO_3_)_3_·6H_2_O and heated to 160 °C for 72 h; colorless, block-shaped crystals of complex **3** in ca. 61.65% based on Sm were obtained. Elemental analysis (%) calculated for C_84_H_56_SmN_9_O_10_ (M = 1501.72): C = 67.18, H = 3.76, N = 8.39; Found: C = 66.89, H = 3.36, N = 7.45. IR (KBr pellets, cm^−1^):3400, 1594, 1500, 1415, 1239, 1163, 870,825, 631 (Figure S6c).

Synthesis of [Eu(cpt)_3_(H_2_O)] (**4**).

Complex **4** was obtained by a similar procedure to that for **1**, except that Eu (NO_3_)_3_·6H_2_O (44.61 mg, 0.1 mmol) was used instead of La(NO_3_)_3_·6H_2_O and heated to 160 °C for 72 h; colorless, block-shaped crystals of complex **4** in ca. 63.51% based on Eu were obtained. Elemental analysis (%) calculated for C_84_H_55_EuN_9_O_10_ (M = 1502.33): C = 67.15, H = 3.69, N = 8.39; Found: C = 66.74, H = 3.25, N = 7.89. IR (KBr pellets, cm^−1^): 3280, 1601, 1527, 1415, 1394, 1239, 1167, 870, 826, 634 (Figure S6d).

Synthesis of [Gd(cpt)_3_(H_2_O)] (**5**).

Complex **5** was obtained by a similar procedure to that for **1**, except that Gd(NO_3_)_3_·6H_2_O (45.14 mg, 0.10 mmol) was used instead of La(NO_3_)_3_·6H_2_O. Colorless, block-shaped crystals of complex **5** in ca. 65.69% based on Gd were obtained. Elemental analysis (%) calculated for C_84_H_56_GdN_9_O_10_ (M = 1508.62): C = 66.87, H = 3.74, N = 8.36; Found: C = 6, H = 3.46, N = 8.14. IR (KBr pellets, cm^−1^): 3260, 1594, 1506, 1393, 1239, 1005, 874, 825, 637 (Figure S6e).

Synthesis of [Dy(cpt)_3_(H_2_O)] (**6**).

Complex **6** was obtained by a similar procedure to that for **1**, except that Dy(NO_3_)_3_·6H_2_O (45.67 mg, 0.1 mmol) was used instead of La(NO_3_)_3_·6H_2_O. Colorless, block-shaped crystals of complex 6 in ca. 67.65% based on Dy were obtained. Elemental analysis (%) calculated for C_84_H_56_DyN_9_O_10_ (M = 1513.87): C = 66.64, H = 3.73, N = 8.33; Found for: C = 65.75, H = 3.38, N = 7.92. IR (KBr pellets, cm^−1^): 3400, 1596, 1498, 1415, 1237, 1008, 870, 824, 630 (Figure S6f).

Synthesis of [Er(cpt)_3_(H_2_O)] (**7**).

Complex **7** was obtained by a similar procedure to that for **1**, except that Er(NO_3_)_3_·6H_2_O (23.07 mg, 0.05 mmol) was used instead of La(NO_3_)_3_·6H_2_O; colorless, block-shaped crystals of complex **7** in ca. 69.57% based on Er were obtained. Elemental analysis (%) calculated for C_84_H_56_ErN_9_O_10_ (M = 1518.63): C = 66.44, H = 3.72, N = 8.30; Found: C = 65.64, H = 3.36, N = 8.12. IR (KBr pellets, cm^−1^): 3360, 1604, 1502, 1426, 1236, 1068, 875, 824, 626 (Figure S6g).

### 3.3. X-ray Crystallography

We selected a single crystal with a clean and smooth surface, no concave surface, and no cracks. At the temperature of 293(2) K, we used a BRUKER SMART APEX-IICCD X-ray single crystal diffractometer and graphite monochromatic Mo-Kα (λ = 0.71073 Å) rays, and diffraction data were collected in ω-φ scanning. All data were corrected by empirical absorption, and the crystal structure was solved using the direct method. The anisotropy parameters and the coordinates of all non-hydrogen atoms were corrected using the least squares method. F^2^ was refined using the SHELXTL-97 program, and the hydrogen atoms were obtained by theoretical calculation methods. Crystallographic data and experimental details of structural analyses for complexes are summarized in Table S1. The selected bond length and angle parameters are listed in Table S2.

## 4. Conclusions

In conclusion, after hydrothermal synthesis, seven new Lanthanide coordination polymers, namely [Ln(cpt)_3_H_2_O)]n(Ln = La (**1**), Pr (**2**), Sm (**3**), Eu (**4**), Gd (**5**), Dy (**6**), and Er (**7**)), were successfully obtained. Remarkably, complex **4** exhibited remarkable sensitivity and selectivity toward Co^2+^, Cu^2+^ and nitrobenzene. Complex **3** demonstrated effective detection of Cu^2+^ and nitrobenzene through the phenomenon of fluorescence quenching. Furthermore, the mechanism of the high selectivity and sensitivity of complex **4** and **3** for NB were also investigated by theoretical studies. The two coordination polymers exhibited much higher fluorescence quenching responses towards NB due to favorable electron and energy transfer mechanisms. The work presented in this study offers valuable insights for the development of sensitive fluorescent recognition sensors with multifunctional applications.

Crystallographic data for the structural analysis were deposited with the Cambridge Crystallographic Data Center, CCDC reference numbers: 2283726 for complex **1**, 2283709 for complex **2**, 2283707 for complex **3**, 2283701 for complex **4**, 2283708 for complex **5**, 2328935 for complex **6**, and 2283727 for complex **7**. These data can be obtained free of charge from The Cambridge Crystallographic Data Centre via www.ccdc.cam.ac.uk/data_request/cif (accessed on 17 June 2024).

## Data Availability

The data presented in this study are available in the article.

## Supplementary Materials

The following supporting information can be downloaded at: https://www.mdpi.com/article/10.3390/molecules29143438/s1, Figure S1: The emission spectra of free ligand (a), complex **1** (b), **2** (c), **5** (d) and **7** (e) at room temperature; Figure S2: Xrd for **1** (a), **2** (b), **3** (c), **4** (d), **5** (e), **6** (f), **7** (g); Figure S3: PXRD patterns of simulated and experimental samples of complex **4**, **3** before and after treatment in ionic aqueous solutions; Figure S4: FT-IR spectra of complex **4**, **3** before and after treatment in ionic aqueous solutions; Figure S5: Hirshfeld surface mapped with dnorm (left), shape index (middle), and curvedness (right) for **1** (a), **2** (b), **3** (c), **5** (d), **6** (e) and **7** (f); Figure S6: The IR spectra for complex **1** (a), **2** (b), **3** (c), **4** (d), **5** (e), **6** (f) and **7** (g); Table S1: Crystallographic data for complexes **1**–**7**; Table S2: Selected bond lengths and angles for complexes **1**–**7**.

## References

[1] Zhao X., Bu X., Wu T., Zheng S.T., Wang L., Feng P. (2013). Selective anion exchange with nanogated isoreticular positive metal-organic frameworks. Nat. Commun..

[2] Boyd S.A., Sheng G., Teppen B.J., Johnston C.T. (2001). Mechanisms for the Adsorption of Substituted Nitrobenzenes by Smectite Clays. Environ. Sci. Technol..

[3] Li Z., Zhan Z., Hu M. (2020). A luminescent terbium coordination polymer as a multifunctional water-stable sensor for detection of Pb^2+^ ions, PO_4_^3−^ ions, Cr_2_O_7_^2−^ ions, and some amino acids. CrystEngComm.

[4] Gahlaut P.S., Gautam D., Yadav K., Barun J. (2023). Supramolecular Gels for the Sensing and Extraction of Heavy Metal Ions from Wastewater. J. Mol. Struct..

[5] Fayed T.A., El-Nahass M.N., El-Daly H.A., Shokry A.A. (2019). Development of nanomaterial chemosensors for toxic metal ions sensing. Appl. Organomet. Chem..

[6] Mandal S., Das A., Biswas A., Halder A., Mondal D., Mondal T.K. (2024). Adaptable Biomolecule-Interactive Dual Colorimetric Chemosensor for Cu^2+^ and Pd^2+^: Insight from Crystal Structure, Photophysical Investigations, Real-Time Sampling, and Molecular Logic Circuits. Cryst. Growth Des..

[7] Senthilkumar S., Goswami R., Smith V.J., Bajaj H.C., Neogi S. (2018). Pore wall-functionalized luminescent Cd (II) framework for selective CO_2_ adsorption, highly specific 2, 4, 6-trinitrophenol detection, and colorimetric sensing of Cu^2+^ ions. ACS Sustain. Chem. Eng..

[8] Huang L., Ran Z., Liu X., Huang C.M., Qin Q.P., Zhou J. (2022). One Luminescent Cadmium Iodide with Free Bifunctional Azole Sites as a Triple Sensor for Cu^2+^, Fe^3+^, and Cr_2_O_7_^2−^ Ions. Inorg. Chem..

[9] Udhayakumari D., Naha S., Velmathi S. (2017). Colorimetric and fluorescent chemosensors for Cu ^2+^. A comprehensive review from the years. Anal. Methods-UK..

[10] Hao Z., Song X., Zhu M., Meng X., Zhao S., Su S., Zhang H. (2013). One-dimensional channel-structured Eu-MOF for sensing small organic molecules and Cu^2+^ ion. J. Mater Chem. A..

[11] Chen L., Cui X., Cheng H., Chen X., Song M., Tang M., Wu Y. (2012). Syntheses, structures of N-(substituted)-2-aza-[3]-ferrocenophanes and their application as redox sensor for Cu^2+^ ion. Appl. Organomet. Chem..

[12] Mohandoss S., Stalin T. (2017). A new fluorescent PET sensor probe for Co^2+^ ion detection: Computational, logic device and living cell imaging applications. RSC Adv..

[13] Khan I.M., Shakya S. (2019). Exploring Colorimetric Real-Time Sensing Behavior of a Newly Designed CT Complex toward Nitrobenzene and Co^2+^: Spectrophotometric, DFT/TD-DFT, and Mechanistic Insights. ACS Omega.

[14] Maity D., Govindaraju T. (2011). Highly selective visible and near-IR sensing of Cu^2+^ based on thiourea-salicylaldehyde coordination in aqueous media. Chemistry.

[15] Vellingiri K., Boukhvalov D.W., Pandey S.K., Deep A., Kim K.H. (2017). Luminescent metal-organic frameworks for the detection of nitrobenzene in aqueous media. Sens. Actuators B Chem..

[16] Li X.Y., Zeng H., Hu H.M., Sun L.J., Zhang J.L., Wang X.F. (2022). Multiterpyridyl Ligand/Cadmium(II) Coordination Polymer Nanosheets for Recoverable Luminescent Sensors. ACS Appl. Nano Mater..

[17] Ruiz-Ramirez M.M., Silva-Carrillo C., Hinostroza-Mojarro J.J., Rivera-Lugo Y.Y., Valle-Trujillo P., Trujillo-Navarrete B. (2021). Electrochemical sensor for determination of nitrobenzene in aqueous solution based on nanostructures of TiO_2_/GO. Fuel.

[18] Zhang J., Deng Y., Wang S., Yang J., Hu S. (2023). A calixarene-based coordination cage as an efficient luminescent sensor for Fe^3+^, MnO_4_^−^, NB and 2, 4-DNP in aqueous medium. CrystEngComm.

[19] Karmakar A., Samanta P., Dutta S., Ghosh S.K. (2019). Fluorescent “turn-on” sensing based on metal–organic frameworks (MOFs). Chem.-Asian J..

[20] Wu J.X., Yan B. (2016). Photofunctional hybrid based lanthanide functionalized metal-organic frameworks by ion exchange and coordination modification for luminescent sensing. Inorg. Chem. Commun..

[21] Li Q., Qian J., Zhou J., Du L., Zhao Q. (2020). Highly chemically and thermally stable lanthanide coordination polymers for luminescent probes and white light emitting diodes. CrystEngComm.

[22] Wu Y., Li M., Liu D., Liu M., Qian J. (2019). Two-dimensional Cd(II) coordination polymer encapsulated by Tb^3+^ as a reversible luminescent probe for Fe^3+^. RSC Adv..

[23] Chen H., Fan P., Tu X., Min H., Yu X., Li X., Cheng P. (2019). A bifunctional luminescent metal–organic framework for the sensing of paraquat and Fe^3+^ ions in water. Chem.-Asian J..

[24] Chen Z., Cai Y., Ma Y., Huang L., Zhao Y., Wang L. (2021). Luminescent lanthanide complex sensor for Acac and Cd^2+^. Photochem. Photobiol..

[25] Gao J.H., Huang P.P., Zhang Z.J., Tian F.W., Ge J., Cao X.Y., Liu J., Wang D., Zheng N., Lu J.F. (2024). A new 3D Cd-MOF with 2fold interpenetrated as “turn-on/turn-off” fluorescent sensor for selective and sensitive detection of Cu^2+^, Al^3+^ and Fe^3+^ ions. J. Mol. Struct..

[26] Wang L., Tu B., Xu W., Fu Y., Zheng Y. (2020). Uranyl Organic Framework as a Highly Selective and Sensitive Turn-on and Turn-off Luminescent Sensor for Dual Functional Detection Arginine and MnO. Inorg. Chem..

[27] Wang X., Xu Q.W., Wei M.M., Chen J.Y., Wang H.H., Li X. (2022). Lanthanide ternary mixed-ligand coordination polymers as fluorescent sensors for the sensitive and selective detection of chlorogenic acid. CrystEngComm.

[28] Xu X.Y., Yan B. (2015). Eu(III)-Functionalized MIL-124 as Fluorescent Probe for Highly Selectively Sensing Ions and Organic Small Molecules Especially for Fe(III) and Fe(II). ACS Appl. Mater. Interfaces.

[29] Lu Z.Z., Zhang R., Li Y.Z., Guo Z., Zheng H.G. (2011). Solvatochromic Behavior of a Nanotubular Metal-Organic Framework for Sensing Small Molecules. J. Am. Chem. Soc..

[30] Mautner F.A., Bierbaumer F., Fischer R.C., Vicente R., Tubau À., Ferran A., Massoud S.S. (2021). Structural Characterization, Magnetic and Luminescent Properties of Praseodymium(III)-4,4,4-Trifluoro-1-(2-Naphthyl)Butane-1,3-Dionato(1-) Complexes. Crystals.

[31] Mautner F.A., Bierbaumer F., Vicente R., Speed S., Tubau Á., Font-Bardía M., Fischer R.C., Massoud S.S. (2022). Magnetic and luminescence properties of 8-coordinate holmium (iii) complexes containing 4, 4, 4-trifluoro-1-phenyl-and 1-(naphthalen-2-yl)-1, 3-butanedionates. Molecules.

[32] Han L.J., Kong Y.J., Zhang X.M., Hou G.Z., Chen H.C., Zheng H.G. (2021). Fabrication of a robust lanthanide metal–organic framework as a multifunctional material for Fe (III) detection, CO_2_ capture, and utilization. J. Mater. Chem. C.

[33] Wei N., Zhang M.Y., Zhang X.N., Li G.M., Zhang X.D., Han Z.B. (2014). Two series of solvent-dependent lanthanide coordination polymers demonstrating tunable luminescence and catalysis properties. Cryst. Growth Des..

[34] Tarlton M.L., Skanthakumar S., Hutchison D., Gremillion A.J., Oliver A.G., Wilson R.E. (2022). Synthesis of an isostructural series of 12-coordinate lanthanide nitrate hybrid double perovskites with cubic symmetry. Inorg. Chem..

[35] Xu W.T., Zhou Y.F., Huang D.C., Wei X., Su M.Y., Wang K., Han S., Hong M.C. (2013). Crystal structure, multiplex photoluminescence, and magnetic properties of a series of lanthanide coordination polymers based on quinoline carboxylate ligand. Cryst. Growth Des..

[36] Jing T., Chen L., Jiang F., Yang Y., Zhou K., Yu M., Cao Z., Li S., Hong M. (2018). Fabrication of a Robust Lanthanide Metal–Organic Framework as a Multifunctional Material for Fe(III) Detection, CO_2_ Capture, and Utilization. Cryst. Growth Des..

[37] Yang Y., Qiu F., Xu C., Feng Y., Zhang G., Liu W.A. (2018). A multifunctional Eu-CP as a recyclable luminescent probe for the highly sensitive detection of Fe^3+^/Fe^2+^, Cr_2_O_7_^2−^, and nitroaromatic explosives. Dalton Trans..

[38] Smith J.A., Singh-Wilmot M.A., Carter K.P., Cahill C.L., Ridenour J.A. (2018). Lanthanide-2, 3, 5, 6-tetrabromoterephthalic acid metal–organic frameworks: Evolution of halogen···halogen interactions across the lanthanide series and their potential as selective bifunctional sensors for the detection of Fe^3+^, Cu^2+^, and nitroaromatics. Cryst. Growth Des..

[39] Zhong X.F., Ma Z.C., Lin J.J., Wu Y., Liang G., Zhang Y.Y., Chen D.J., Xie K.P., Mo Z.W., Chen X.M. (2023). Metal–Organic Frameworks with Triazine and Amine Functional Groups for Iodine Removal and Sensitive Detection of Cu^2+^ and Fe^3+^ Ions. Cryst. Growth Des..

[40] Cui Y., Yue Y., Qian G., Chen B. (2012). Luminescent functional metal-organic frameworks. Chem. Rev..

[41] Kreno L.E., Leong K., Farha O.K., Allendorf M., VanDuyne R., Hupp J.T. (2012). Metal–organic framework materials as chemical sensors. Chem. Rev..

[42] Liu B. (2012). Metal–organic framework-based devices: Separation and sensors. J. Mater. Chem..

[43] Banerjee D., Hu Z., Li J. (2014). Luminescent metal–organic frameworks as explosive sensors. Dalton Trans..

[44] Cui Y., Chen B., Qian G. (2014). Lanthanide metal-organic frameworks for luminescent sensing and light-emitting applications. Chem. Rev..

[45] Hu Z., Deibert B.J., Li J. (2014). Luminescent metal–organic frameworks for chemical sensing and explosive detection. Chem. Soc. Rev..

[46] Nagarkar S.S., Desai A.V., Ghosh S.K. (2016). Engineering metal–organic frameworks for aqueous phase 2, 4, 6-trinitrophenol (TNP) sensing. CrystEngComm.

[47] Lustig W.P., Mukherjee S., Rudd N.D., Desai A.V., Li J., Ghosh S.K. (2017). Metal–organic frameworks: Functional luminescent and photonic materials for sensing applications. Chem. Soc. Rev..

[48] Nagarkar S.S., Joarder B., Chaudhari A.K., Mukherjee S., Ghosh S.K. (2013). Highly selective detection of nitro explosives by a luminescent metal-organic framework. Chem. Int. Ed..

[49] Nagarkar S.S., Desai A.V., Ghosh S.K. (2014). A fluorescent metal–organic framework for highly selective detection of nitro explosives in the aqueous phase. Chem. Commun..

[50] Joarder B., Desai A.V., Samanta P., Mukherjee S., Ghosh S.K. (2015). Selective and sensitive aqueous-phase detection of 2, 4, 6-trinitrophenol (TNP) by an amine-functionalized metal–organic framework. Chem. Eur. J..

[51] Mukherjee S., Desai A.V., Inamdar A.I., Manna B., Ghosh S.K. (2015). Selective detection of 2, 4, 6-trinitrophenol (TNP) by a π-stacked organic crystalline solid in water. Cryst. Growth Design.

[52] Mukherjee S., Desai A.V., Manna B., Inamdar A.I., Ghosh S.K. (2015). Exploitation of guest accessible aliphatic amine functionality of a metal–organic framework for selective detection of 2, 4, 6-trinitrophenol (TNP) in water. Cryst. Growth Des..

[53] Nagarkar S.S., Desai A.V., Samanta P., Ghosh S.K. (2015). Aqueous phase selective detection of 2, 4, 6-trinitrophenol using a fluorescent metal–organic framework with a pendant recognition site. Dalton Trans..

[54] Karmakar A., Kumar A., Chaudhari A.K., Samanta P., Desai A.V., Krishna R., Ghosh S.K. (2016). Bimodal Functionality in a Porous Covalent Triazine Framework by Rational Integration of an Electron-Rich and-Deficient Pore Surface. Chem.-Eur. J..

[55] Surya S.G., Nagarkar S.S., Ghosh S.K., Sonar P., Rao V.R. (2016). OFET based explosive sensors using diketopyrrolopyrrole and metal organic framework composite active channel material. Sens. Actuators. B..

[56] Liu X.G., Wang H., Chen B., Zou Y., Gu Z.G., Zhao Z., Shen L. (2015). A luminescent metal–organic framework constructed using a tetraphenylethene-based ligand for sensing volatile organic compounds. Chem. Commun..

[57] Wang G.Y., Song C., Kong D.M., Ruan W.J., Chang Z., Li Y. (2014). Two luminescent metal–organic frameworks for the sensing of nitroaromatic explosives and DNA strands. J. Mater. Chem. A.

[58] Tian H.R., Gao C.Y., Yang Y., Ai J., Liu C., Xua Z.G., Sun Z.M. (2017). A microporous Cd-MOF based on a hexavalent silicon-centred connector and luminescence sensing of small molecules. New J. Chem..

[59] Wang L., Xie Z.G., Dang S., Sun Z.M. (2017). Self-Assembly of Tunable Heterometallic Ln–Ru Coordination Polymers with Near-Infrared Luminescence and Magnetocaloric Effect. Chem.-Eur. J..

[60] Buragohain A., Yousufuddin M., Sarma M., Biswas S. (2016). 3D luminescent amide-functionalized cadmium tetrazolate framework for selective detection of 2, 4, 6-trinitrophenol. Cryst. Growth Des..

[61] Huang R.W., Wei Y.S., Dong X.Y., Wu X.H., Du C.X., Zang S.Q., Mak T.C.W. (2017). Hypersensitive dual-function luminescence switching of a silver-chalcogenolate cluster-based metal–organic framework. Nat. Chem..

[62] Yang L., Cao L., Li X., Qin C., Zhao L., Shao K.Z., Su Z.M. (2017). Metal–organic frameworks constructed from tib and carboxylate acid ligands: Selective sensing of nitro explosives and magnetic properties. Dalton Trans..

[63] Gole B., Bar A.K., Mukherjee P.S. (2014). Modification of extended open frameworks with fluorescent tags for sensing explosives: Competition between size selectivity and electron deficiency. Chem.-Eur. J..

[64] Gao J.H., Wang J.X., Huang P.P., Liu J., Zheng N., Shi J., Xu H.T., Yue S.Y., Lu J.F. (2023). A new pyrazine carboxyl derivative and its two d10 metal coordination polymers: Syntheses, characterization, DFT and property. J. Mol. Struct..

[65] Elantabli F.M., Mohamed R.G., El-Medani S.M., Haukka M., Ramadan R.M., Afifi M.A. (2024). Structural investigations of new tridentate-phenylacetohydrazide Schiff base metal chelates: X-ray diffraction, Hirshfeld surface analyses, DFT, antibacterial and molecular docking studies. J. Mol Struct..

[66] Najafi Z., Marandi F., Bahrami A., Fuhrmann D., Janghouri M. (2023). Four new Zn (II) complexes based on 2-thienoyltrifluoroacetone and N-donor auxiliary bridging and chelating ligands: Synthesis, spectroscopic and structural studies, thermal behavior and Hirshfeld surface analysis. Polyhedron.

