# Supplementary material for: A Series of Lanthanide Coordination Polymers as Luminescent Sensors for Selective Detection of Inorganic Ions and Nitrobenzene
Source: Molecules. 2024 Jul 22;29(14):3438. doi: 10.3390/molecules29143438 (PMC11279550; doi:10.3390/molecules29143438)
Supplement: Supplementary file 1 [file molecules-29-03438-s001.zip › molecules-3087932-supplementary.pdf]

.

TABLE S1 Crystallographic data for complexes 1-7

Complex 1			
Formula	$C_{84}H_{56}LaN_9O_{10}$	T/k	293
Formula weight	1490.28	h	12
Crystal system	triclinic	k	19
Space group	P-1	l	23
Color	yellow	F(000)	1520
a/nm	10.7792(4)	Dc/(g.cm^{-3})	1.444
b/nm	16.2179(4)	μ/mm^{-1}	5.398
c/nm	19.8816(5)	θrange/(°)	2.733 to 67.249
α/(°)	85.965(2)	Reflections collected	10297/12114
Β/(°)	81.451(3)	Data / restraints / parameters	12114/0/937
Γ/(°)	87.574(3)	Final R indices [I>2σ（I）]	R1= 0.0404，wR2= 0.0946
V/nm^3	3426.70(18)	R（all data）	R1= 0.0494，wR2= 0.0990
Z	2	GOOF	0.978
λ	1.54184	R_{int}	0.0492
Complex 2			
Formula	$C_{84}H_{56}Pr N_9 O_{10}$	T/k	293
Formula weight	1492.29	h	12
Crystal system	Triclinic	k	18
Space group	P -1	l	22
Color	yellow	F(000)	1524
a/nm	10.7887(2)	Dc/(g.cm^{-3})	1.448
b/nm	16.1941(4)	μ/mm^{-1}	6.788
c/nm	19.8586(4)	θrange/(°)	2.737 to 67.075
α/(°)	85.963(2)	Reflections collected	10992/12086
Β/(°)	81.705(2)	Data / restraints / parameters	12086/0/938
Γ/(°)	87.251(2)	Final R indices [I>2σ（I）]	R1= 0.0378，wR2= 0.0890
V/nm^3	3422.23(13)	R（all data）	R1= 0.0428，wR2= 0.0912
Z	2	GOOF	1.035
λ	1.54184	R_{int}	0.0652
Complex 3			
Formula	$C_{84}H_{56}Sm N_9 O_{10}$	T/k	293
Formula weight	1501.72	h	12
Crystal system	triclinic	k	19
Space group	P -1	l	23
Color	colourless	F(000)	1530
a/nm	10.8010(2)	Dc/(g.cm^{-3})	1.457
b/nm	16.1764(4)	μ/mm^{-1}	7.021

c/nm	19.8794(4)	θrange/(°)	2.742 to 67.072
α/(°)	85.7794(17)	Reflections collected	11036/ 12162
Β/(°)	81.6506(17)	Data / restraints / parameters	12162/0/937
Γ/(°)	86.7790(18)	Final R indices [I>2σ（I）]	R1=0.0320，wR2=0.0704
V/nm^3	3423.58(13)	R（all data）	R1=0.0372，wR2=0.0730
Z	2	GOOF	1.029
λ	1.54184	R_{int}	0.0582
	Complex 4		
Formula	$C_{84}H_{55}EuN_9O_{10}$	T/k	293
Formula weight	1502.33	h	12
Crystal system	triclinic	k	19
Space group	P -1	l	23
Color	colourless	F(000)	1530
a/nm	10.8073(2)	Dc/(g.cm^{-3})	1.459
b/nm	16.1735(3)	μ/mm^{-1}	7.144
c/nm	19.8549(3)	θrange/(°)	2.743 to 67.077
α/(°)	85.724(2)	Reflections collected	11346/12096
Β/(°)	81.674(1)	Data / restraints / parameters	12096/4/943
Γ/(°)	86.736(2)	Final R indices [I>2σ（I）]	R1=0.0347，wR2=0.0796
V/nm^3	3420.59(10)	R（all data）	R1=0.0380，wR2=0.0808
Z	2	GOOF	1.042
λ	1.54184	R_{int}	0.0531
	Complex 5		
Formula	$C_{84}H_{56}GdN_9O_{10}$	T/k	293
Formula weight	1508.62	h	12
Crystal system	triclinic	k	19
Space group	P -1	l	23
Color	colourless	F(000)	1530
a/nm	10.8001(6)	Dc/(g.cm^{-3})	1.451
b/nm	16.288(2)	μ/mm^{-1}	6.788
c/nm	19.8506(6)	θrange/(°)	4.140 to 67.073
α/(°)	90.246(7)	Reflections collected	10426/12128
Β/(°)	98.162(3)	Data / restraints / parameters	12128/4/938
Γ/(°)	92.695(8)	Final R indices [I>2σ（I）]	R1= 0.0717，wR2= 0.2027
V/nm^3	3452.6(5)	R（all data）	R1= 0.0914，wR2= 0.2362
Z	2	GOOF	1.081
λ	1.54184	R_{int}	0.1280
	Complex 6		

Formula	$C_{84}H_{56}DyN_9O_{10}$	T/k	273
Formula weight	1513.87	h	13
Crystal system	triclinic	k	20
Space group	P-1	l	25
Color	yellow	F(000)	1538
a/nm	10.7892(15)	Dc/(g.cm^{-3})	1.477
b/nm	16.153(2)	μ/mm^{-1}	1.169
c/nm	19.820(3)	θrange/(°)	1.041 to 27.161
α/(°)	85.585(4)	Reflections collected	9224/14816
Β/(°)	81.732(3)	Data / restraints / parameters	14816/3/937
Γ/(°)	86.708(4)	Final R indices [I>2σ（I）]	R1= 0.0720，wR2=0.1621
V/nm^3	3404.3(8)	R（all data）	R1= 0.1300，wR2=0.1987
Z	2	GOOF	1.035
λ	0.71073	R_{int}	0.0660
	Complex 7		
Formula	$C_{84}H_{56}ErN_9O_{10}$	T/k	298
Formula weight	1518.63	h	12
Crystal system	Triclinic	k	19
Space group	P -1	l	23
Color	yellow	F(000)	1542
a/nm	10.8070(2)	Dc/(g.cm^{-3})	1.477
b/nm	16.1667(3)	μ/mm^{-1}	2.864
c/nm	19.8287(3)	Θrange/(°)	2.745 to 67.077
α/(°)	85.4080(10)	Reflections collected	10847/12092
Β/(°)	81.7450(10)	Data / restraints / parameters	12092/0/938
Γ/(°)	86.6990(10)	Final R indices [I>2σ（I）]	R1= 0.0297，wR2=0.0755
V/nm^3	3413.71(10)	R（all data）	R1= 0.0345，wR2=0.0777
Z	2	GOOF	1.026
λ	1.54184	R_{int}	0.0445

TABLE S2. Selected bond lengths (Å) and angles (°) for complexes 1–7

Complex 1			
La(1)- O(1)	2.512(2)	La(1)- O(10)	2.519(3)
La(1)- O(8)	2.520(3)	La(1)- O(4)	2.521(2)
La(1)- O(7)	2.531(2)	La(1)- O(5)	2.537(2)
La(1)- O(2)	2.605(3)	La(1)- O(4)	2.738(2)
La(1)- N(4)	2.772(3)		
O(1)- La(1)- O(10)	138.42(10)	O(1)- La(1)- O(8)	74.42(10)
O(10)- La(1)- O(8)	73.59(10)	O(1)- La(1)- O(4)	80.45(8)
O(10)- La(1)- O(4)	73.18(8)	O(8)- La(1)- O(4)	89.06(8)
O(1)- La(1)- O(7)	101.60(8)	O(10)- La(1)- O(7)	78.02(9)
O(8)- La(1)- O(7)	51.41(8)	O(4)- La(1)- O(7)	136.53(8)
O(1)- La(1)- O(5)	143.04(10)	O(10)- La(1)- O(5)	78.36(10)
O(8)- La(1)- O(5)	134.15(9)	O(4)- La(1)- O(5)	116.52(7)
O(7)- La(1)- O(5)	87.95(8)	O(1)- La(1)- O(2)	50.89(8)
O(10)- La(1)- O(2)	143.02(8)	O(8)- La(1)- O(2)	124.56(9)
O(4)- La(1)- O(2)	75.17(8)	O(7)- La(1)- O(2)	138.96(8)
O(5)- La(1)- O(2)	99.50(9)	O(1)- La(1)- O(4)	123.30(7)
O(10)- La(1)- O(4)	76.31(9)	O(8)- La(1)- O(4)	146.99(9)
O(4)- La(1)- O(4)	69.36(8)	O(7)- La(1)- O(4)	133.36(7)
O(5)- La(1)- O(4)	49.10(7)	O(2)- La(1)- O(4)	75.01(7)
O(1)- La(1)- N(4)	79.13(9)	O(10)- La(1)- N(4)	136.86(9)
O(8)- La(1)- N(4)	109.26(9)	O(4)- La(1)- N(4)	147.51(9)
O(7)- La(1)- N(4)	72.73(9)	O(5)- La(1)- N(4)	69.71(9)
O(2)- La(1)- N(4)	72.34(8)	O(4)- La(1)- N(4)	101.86(8)
La(1)- O(4)- La(1)	110.64(8)		
Complex 2			
Pr (1)- O(1)	2.707(2)	Pr (1)- O(1)	2.4909(18)
Pr (1)- O(2)	2.555(2)	Pr (1)- O(3)	2.491(2)
Pr (1)- O(4)	2.474(2)	Pr (1)- O(6)	2.489(2)
Pr (1)- O(7)	2.483(2)	Pr (1)- O(9)	2.475(2)
Pr (1)- N(3)	2.742(2)		
O(1)- Pr(1)- O(1)	68.83(7)	O(1)- Pr(1)- O(2)	75.23(7)
O(1)- Pr(1)- N(3)	102.69(7)	O(1)- Pr(1)- N(3)	147.72(7)
O(2)- Pr(1)- O(1)	74.66(7)	O(2)- Pr(1)- N(3)	72.49(7)
O(3)- Pr(1)- O(1)	136.38(7)	O(3)- Pr(1)- O(1)	132.82(7)
O(3)- Pr(1)- O(2)	139.91(7)	O(3)- Pr(1)- N(3)	72.84(7)
O(4)- Pr(1)- O(1)	80.92(7)	O(4)- Pr(1)- O(1)	123.79(7)
O(4)- Pr(1)- O(2)	51.84(8)	O(4)- Pr(1)- O(3)	101.86(8)
O(4)- Pr(1)- O(6)	143.02(9)	O(4)- Pr(1)- O(7)	73.59(9)
O(4)- Pr(1)- O(9)	138.67(9)	O(4)- Pr(1)- N(3)	78.82(8)
O(6)- Pr(1)- O(1)	116.57(7)	O(6)- Pr(1)- O(1)	49.58(6)
O(6)- Pr(1)- O(2)	98.96(8)	O(6)- Pr(1)- O(3)	87.36(8)
O(6)- Pr(1)- N(3)	69.78(8)	O(7)- Pr(1)- O(1)	88.55(7)

O(7)- Pr(1)- O(1)	146.44(8)	O(7)- Pr(1)- O(2)	124.54(8)
O(7)- Pr(1)- O(3)	52.32(7)	O(7)- Pr(1)- O(6)	134.80(9)
O(7)- Pr(1)- N(3)	109.24(8)	O(9)- Pr(1)- O(1)	73.56(7)
O(9)- Pr(1)- O(1)	76.09(8)	O(9)- Pr(1)- O(2)	143.11(7)
O(9)- Pr(1)- O(3)	76.97(8)	O(9)- Pr(1)- O(6)	78.21(10)
O(9)- Pr(1)- O(7)	73.80(10)	O(9)- Pr(1)- N(3)	136.40(8)
Pr(1)- O(1)- Pr(1)	111.17(7)		
Complex 3			
Sm(1)- O(1)	2.4919(19)	Sm(1)- O(4)	2.4282(19)
Sm(1)- O(6)	2.4494(18)	Sm(1)- O(8)	2.4515(19)
Sm(1)- O(10)	2.426(2)	Sm(1)- O(5)	2.435(2)
Sm(1)- O(3)	2.4449(18)	Sm(1)- O(3)	2.7213(19)
Sm(1)- N(1)	2.724(2)		
O(1)- Sm(1) -O(3)	74.30(6)	O(1)- Sm(1) -N(1)	72.36(7)
O(4)- Sm(1) -O(1)	96.81(7)	O(4)- Sm(1) -O(6)	89.18(7)
O(4)- Sm(1) -O(8)	136.49(8)	O(4)- Sm(1) -O(5)	141.21(8)
O(4)- Sm(1) -O(3)	49.97(6)	O(4)- Sm(1) -O(3)	117.72(7)
O(1)- Sm(1) -N(1)	68.99(8)	O(6)- Sm(1) -O(1)	139.58(7)
O(6)- Sm(1) -O(8)	53.12(6)	O(6)- Sm(1) -O(3)	134.16(6)
O(6)- Sm(1) -N(1)	72.74(7)	O(8)- Sm(1) -O(1)	125.53(7)
O(10)- Sm(1) -O(1)	142.98(6)	O(10)- Sm(1) -O(4)	77.92(9)
O(10)- Sm(1) -O(6)	77.37(7)	O(10)- Sm(1) -O(8)	73.55(8)
O(10)- Sm(1) -O(5)	140.82(8)	O(10)- Sm(1) -O(3)	74.63(7)
O(10)- Sm(1) -O(3)	74.62(7)	O(10)- Sm(1) -N(1)	135.07(7)
O(5)- Sm(1) -O(1)	52.94(6)	O(5)- Sm(1) -O(6)	99.98(7)
O(5)- Sm(1) -O(8)	74.10(8)	O(5)- Sm(1) -O(3)	124.48(6)
O(5)- Sm(1) -O(3)	81.35(7)	O(5)- Sm(1) -N(1)	77.89(7)
O(3)- Sm(1) -O(1)	75.86(6)	O(3)- Sm(1) -O(6)	135.15(6)
O(3)- Sm(1) -O(8)	85.42(6)	O(3)- Sm(1) -O(3)	69.13(6)
O(3)- Sm(1) -N(1)	103.77(6)	O(3)- Sm(1) -N(1)	148.14(7)
Sm(1)- O(3)- Sm(1)	110.88(6)		
Complex 4			
Eu(1)-O(1)	2.4277(18)	Eu(1)-O(10)	2.426(2)
Eu(1)-O(1)	2.725(2)	Eu(1)-O(7)	2.4090(19)
Eu(1)-O(4)	2.481(2)	Eu(1)-O(2)	2.409(2)
Eu(1)-O(6)	2.4360(19)	Eu(1)-N(4)	2.715(2)
Eu(1)-O(8)	2.443(2)		
O(1)-Eu(1)-O(1)	68.87(6)	O(1)-Eu(1)-O(4)	75.99(6)
O(1)-Eu(1)-O(6)	134.98(6)	O(1)-Eu(1)-O(8)	85.16(7)
O(1)-Eu(1)-N(4)	148.30(7)	O(4)-Eu(1)-O(1)	74.00(6)
O(4)-Eu(1)-N(4)	72.41(7)	O(6)-Eu(1)-O(1)	134.37(6)
O(6)-Eu(1)-O(4)	139.76(7)	O(6)-Eu(1)-O(8)	53.22(6)
O(4)-Eu(1)-N(4)	72.72(7)	O(8)-Eu(1)-O(1)	143.11(7)
O(8)-Eu(1)-O(4)	125.77(7)	O(8)-Eu(1)-N(4)	111.40(8)

O(10)-Eu(1)-O(1)	81.53(7)	O(10)-Eu(1)-O(1)	124.44(6)
O(10)-Eu(1)-O(4)	53.27(7)	O(10)-Eu(1)-O(6)	99.88(7)
O(10)-Eu(1)-O(8)	74.10(8)	O(10)-Eu(1)-N(4)	77.72(7)
O(7)-Eu(1)-O(1)	49.94(6)	O(7)-Eu(1)-O(1)	117.53(7)
O(7)-Eu(1)-O(4)	96.07(8)	O(7)-Eu(1)-O(6)	89.66(7)
O(7)-Eu(1)-O(8)	137.07(8)	O(7)-Eu(1)-O(10)	140.81(8)
O(7)-Eu(1)-O(2)	77.81(9)	O(7)-Eu(1)-N(4)	69.00(8)
O(2)-Eu(1)-O(1)	74.80(7)	O(2)-Eu(1)-O(1)	74.33(7)
O(2)-Eu(1)-O(4)	142.82(7)	O(2)-Eu(1)-O(6)	77.31(7)
O(2)-Eu(1)-O(8)	73.82(9)	O(2)-Eu(1)-O(10)	141.33(8)
O(2)-Eu(1)-N(4)	134.73(8)	N(4)- Eu(1)- O(1)	104.14(7)
Eu(1)- O(1)- Eu(1)	111.13(6)		
		Complex 5	
Gd(1)- O(1)	2.383(5)	Gd (1)- O(2)	2.470(5)
Gd (1)- O(3)	2.802(6)	Gd (1)- O(3)	2.346(5)
Gd (1)- O(4)	2.478(6)	Gd (1)- O(5)	2.472(6)
Gd (1)- O(6)	2.503(6)	Gd (1)- O(8)	2.411(7)
Gd (1)- N(4)	2.621(9)		
O(1)-Gd(1)-O(2)	140.0(2)	O(1)- Gd(1)- O(3)	133.02(18)
O(1)- Gd(1)- O(4)	91.5(2)	O(1)- Gd(1)- O(5)	53.05(19)
O(1)- Gd(1)- O(6)	99.1(2)	O(1)- Gd(1)- O(8)	76.5(2)
O(1)- Gd(1)- N(4)	74.4(2)	O(2)- Gd(1)- O(3)	75.44(18)
O(2)- Gd(1)- O(4)	95.8(2)	O(2)- Gd(1)- O(5)	124.4(2)
O(2)- Gd(1)- O(6)	53.74(19)	O(2)- Gd(1)- N(4)	71.37(19)
O(3)- Gd(1)- O(1)	133.0(2)	O(3)- Gd(1)- O(2)	76.69(19)
O(3)- Gd(1)- O(3)	71.6(2)	O(3)- Gd(1)- O(4)	117.4(2)
O(3)- Gd(1)- O(5)	83.6(2)	O(3)- Gd(1)- O(6)	81.1(2)
O(3)- Gd(1)- O(8)	76.3(2)	O(4)- Gd(1)- O(3)	46.95(17)
O(3)- Gd(1)- N(4)	147.9(2)	Gd(1)- O(3)-Gd(1)	108.4(2)
O(4)- Gd(1)- O(6)	141.7(2)	O(4)- Gd(1)- N(4)	70.1(2)
O(5)- Gd(1)- O(3)	144.0(2)	O(5)- Gd(1)- O(4)	138.9(2)
O(5)- Gd(1)- O(6)	72.2(2)	O(5)- Gd(1)- N(4)	111.6(2)
O(6)- Gd(1)- O(3)	126.62(17)	O(6)- Gd(1)- N(4)	77.4(2)
O(8)- Gd(1)- O(2)	143.29(19)	O(8)- Gd(1)- O(3)	72.7(2)
O(8)- Gd(1)- O(4)	75.4(3)	O(8)- Gd(1)- O(5)	76.4(2)
O(8)- Gd(1)- O(6)	142.9(2)	O(8)- Gd(1)- N(4)	133.6(2)
		Complex 6	
Dy(1)- O(7)	2.349(6)	Dy(1)- O(1)	2.354(5)
Dy(1)- O(6)	2.357(5)	Dy(1)- O(4)	2.388(5)
Dy(1)- O(2)	2.394(5)	Dy(1)- O(3)	2.402(6)
Dy(1)- O(5)	2.441(5)	Dy(1)- N(7)	2.689(6)
O(7)- Dy(1)- O(1)	78.0(2)	O(7)- Dy(1)- O(6)	116.95(19)
O(1)- Dy(1)- O(6)	74.91(18)	O(7)- Dy(1)- O(4)	138.7(2)
O(1)- Dy(1)- O(4)	143.0(2)	O(6)- Dy(1)- O(4)	81.52(19)

O(7)- Dy(1)- O(2)	92.4(2)	O(1)- Dy(1)- O(2)	77.96(18)
O(6)- Dy(1)- O(2)	133.93(17)	O(4)- Dy(1)- O(2)	99.64(19)
O(7)- Dy(1)- O(3)	140.4(2)	O(1)- Dy(1)- O(3)	74.6(2)
O(6)- Dy(1)- O(3)	82.69(19)	O(4)- Dy(1)- O(3)	74.5(2)
O(2)- Dy(1)- O(3)	54.32(18)	O(7)- Dy(1)- O(5)	92.4(2)
O(1)- Dy(1)- O(5)	140.83(17)	O(6)- Dy(1)- O(5)	76.10(17)
O(4)- Dy(1)- O(5)	54.51(17)	O(2)- Dy(1)- O(5)	140.91(18)
O(3)- Dy(1)- O(5)	126.69(19)	O(7)- Dy(1)- N(7)	68.7(2)
O(1)- Dy(1)- N(7)	133.95(19)	O(6)- Dy(1)- N(7)	148.91(19)
O(4)- Dy(1)- N(7)	77.5(2)	O(2)- Dy(1)- N(7)	72.75(18)
O(3)- Dy(1)- N(7)	112.9(2)	O(5)- Dy(1)- N(7)	73.07(18)
O(7)- Dy(1)- O(6)	49.30(16)	O(1)- Dy(1)- O(6)	72.57(17)
O(6)- Dy(1)- O(6)	68.44(18)	O(4)- Dy(1)- O(6)	124.07(17)
O(2)- Dy(1)- O(6)	135.27(17)	O(3)- Dy(1)- O(6)	140.68(19)
O(5)- Dy(1)- O(6)	72.60(16)	N(7)- Dy(1)- O(6)	105.35(17)
Dy(1)- O(6)- Dy(1)	111.56(18)		
	Complex 7		
Er(1)- O(1)	2.4132(15)	Er(1)- O(4)	2.3191(17)
Er(1)- O(6)	2.3679(16)	Er(1)- O(8)	2.3628(16)
Er(1)- O(11)	2.3929(17)	Er(1)- O(3)	2.3179(17)
Er(1)- O(7)	2.3449(17)	Er(1)- N(1)	2.6704(19)
O(1)- Er(1)- N(1)	73.53(5)	O(4)- Er(1)- O(1)	76.15(6)
O(4)- Er(1)- O(6)	133.44(6)	O(4)- Er(1)- O(8)	81.74(6)
O(4)- Er(1)- O(11)	81.86(6)	O(4)- Er(1)- O(7)	75.07(6)
O(4)- Er(1)- N(1)	149.35(6)	O(6)- Er(1)- O(1)	141.82(6)
O(6)- Er(1)- O(11)	54.54(6)	O(6)- Er(1)- N(1)	72.85(6)
O(8)- Er(1)- O(1)	54.75(6)	O(8)- Er(1)- O(6)	100.18(6)
O(8)- Er(1)- O(11)	75.78(7)	O(8)- Er(1)- N(1)	77.27(6)
O(11)- Er(1)- O(1)	127.81(6)	O(11)- Er(1)- N(1)	113.71(7)
O(3)- Er(1)- O(1)	89.67(6)	O(3)- Er(1)- O(4)	115.18(6)
O(3)- Er(1)- O(6)	94.95(6)	O(3)- Er(1)- O(8)	137.10(7)
O(3)- Er(1)- O(11)	142.34(7)	O(3)- Er(1)- O(7)	78.17(7)
O(3)- Er(1)- N(1)	69.28(7)	O(7)- Er(1)- O(1)	139.97(6)
O(7)- Er(1)- O(6)	77.75(6)	O(7)- Er(1)- O(8)	144.23(7)
O(7)- Er(1)- O(11)	74.30(7)	O(7)- Er(1)- N(1)	133.30(6)

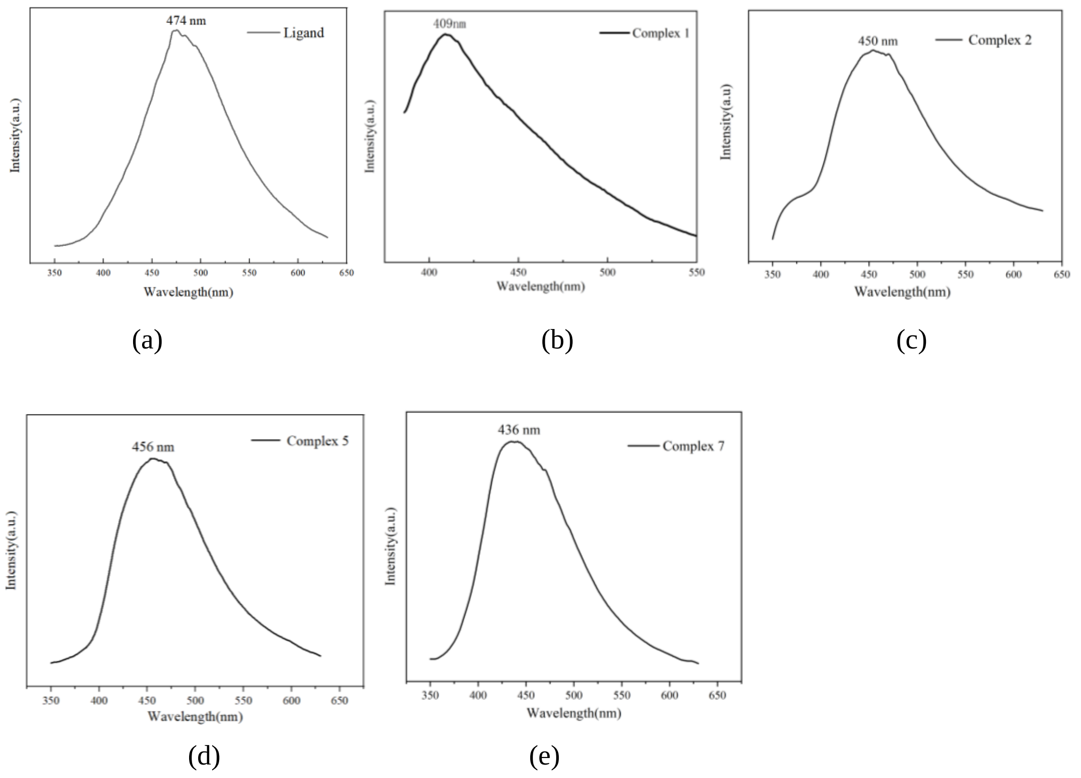

FIGURE S1 The emission spectra of free ligand (a) ,complex 1 (b) , 2 (c) , 5 (d) and 7(e) at room temperature

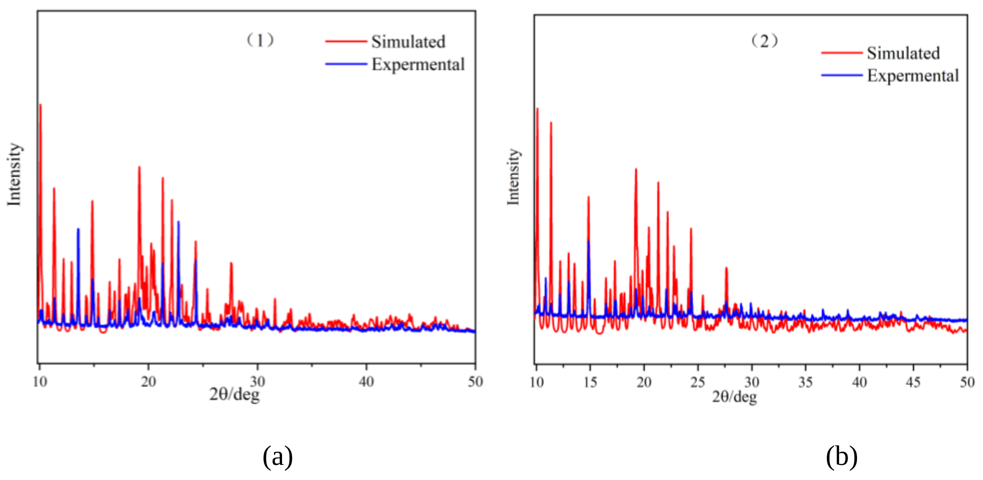

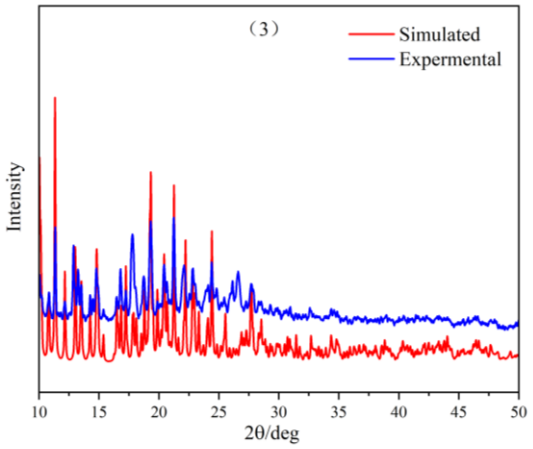

(c)

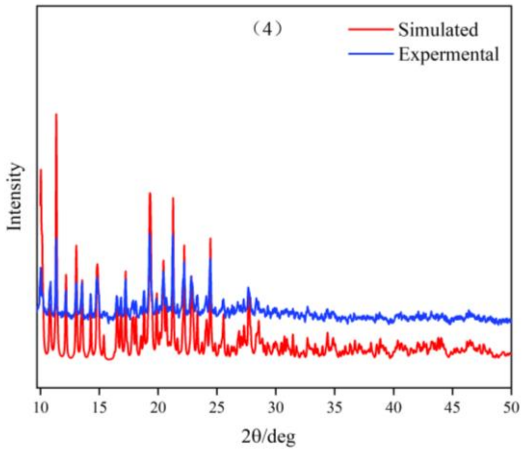

(d)

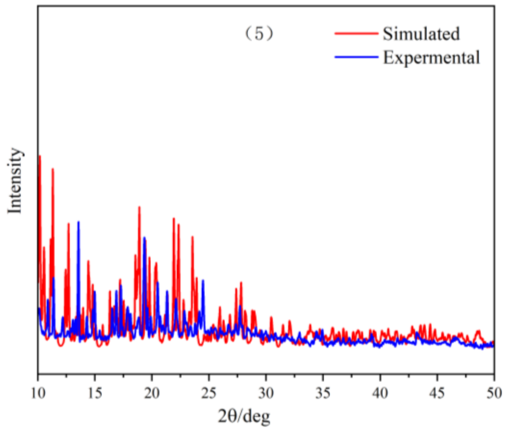

(e)

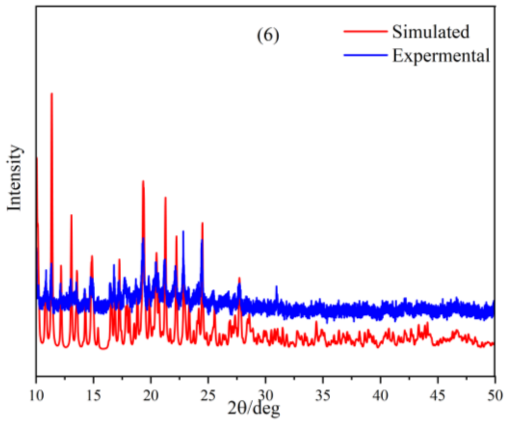

(f)

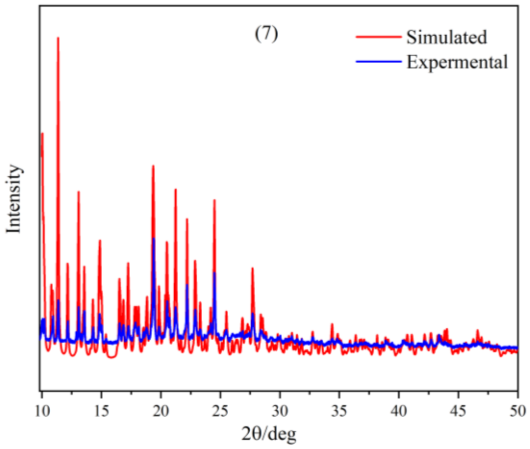

(g)

FIGURE S2 Xrd for 1(a), 2 (b), 3(c),4 (d), 5(e), 6(f), 7(g).

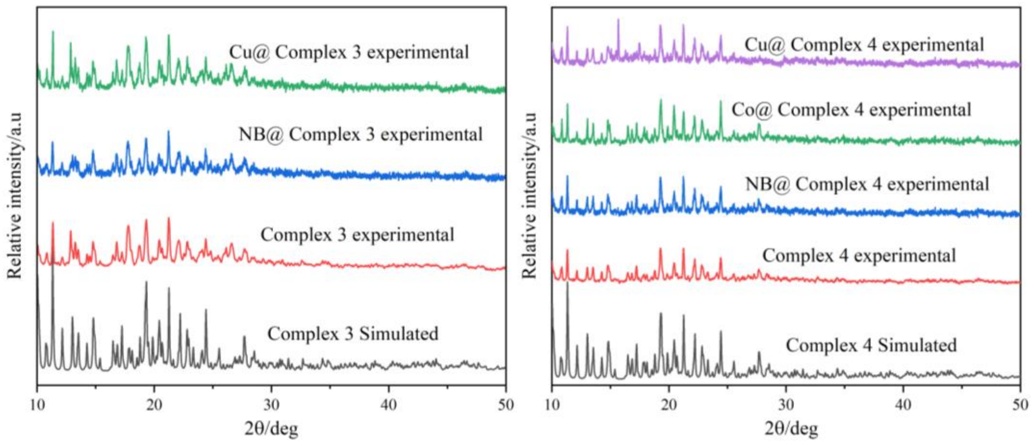

FIGURE S3 . PXRD patterns of simulated and experimental samples of complex **4**, **3** before and after treatment in ionic aqueous solutions

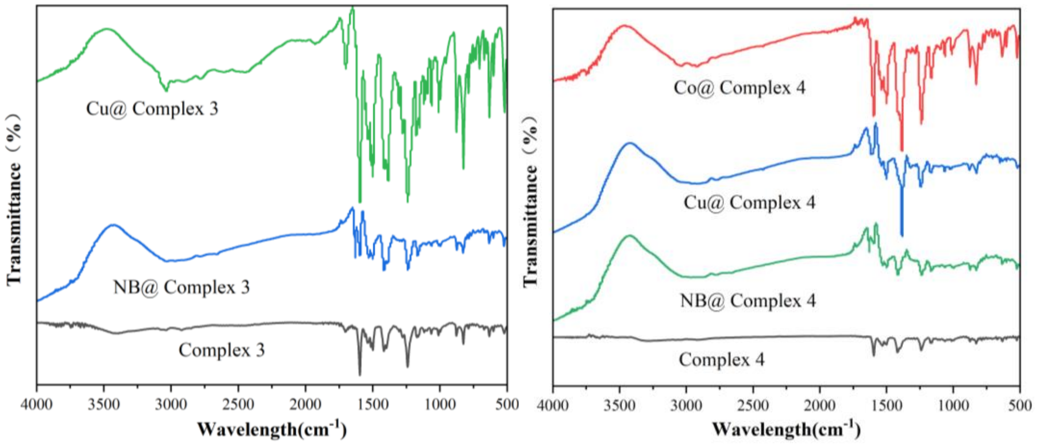

FIGURE S4 . FT-IR spectra of complex 4, 3 before and after treatment in ionic aqueous solutions

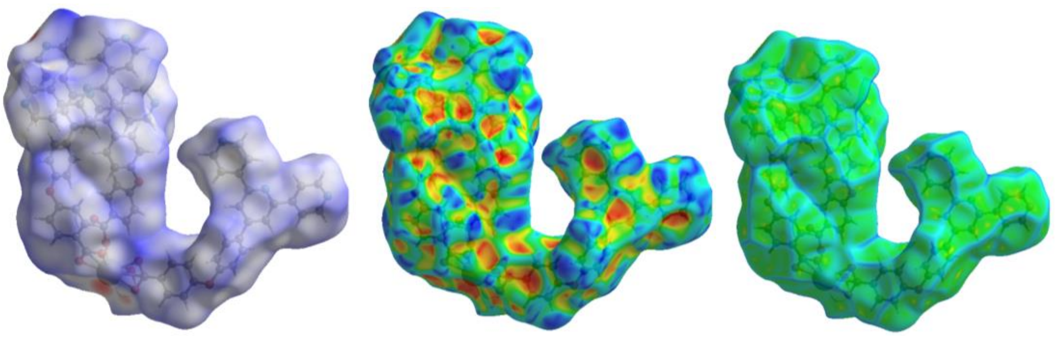

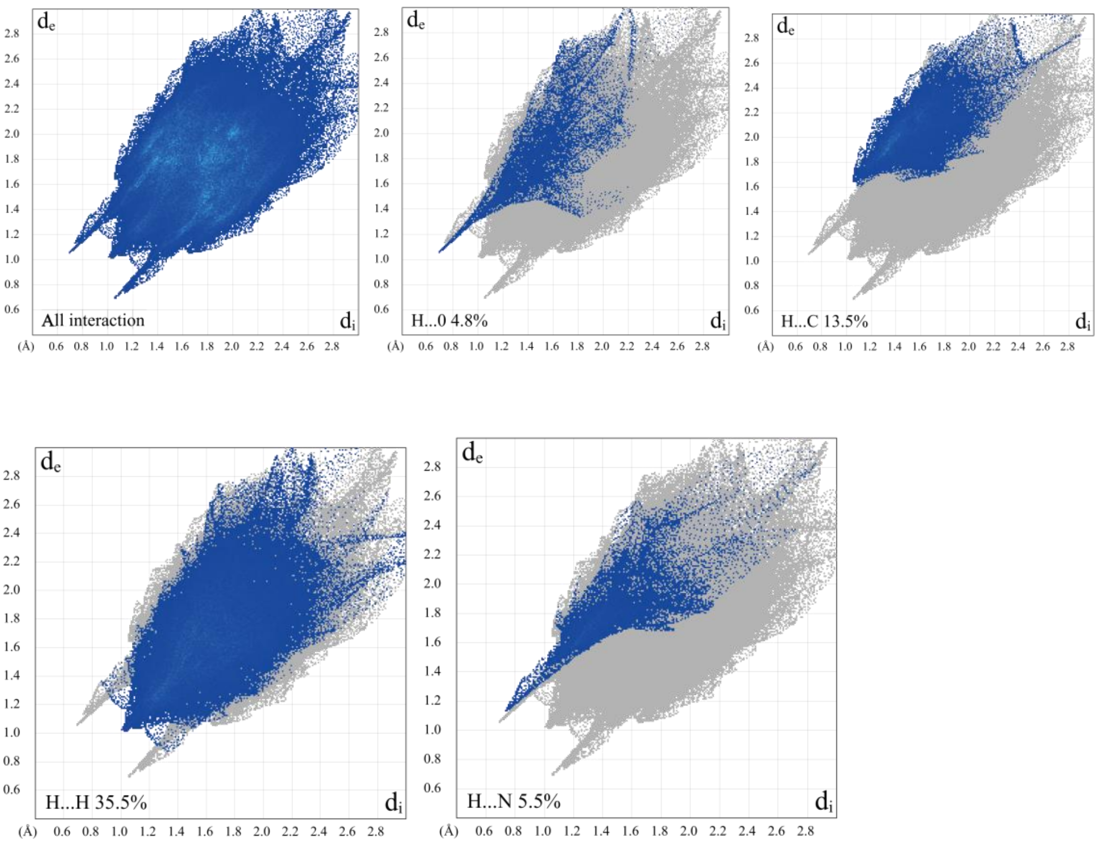
All interaction
H...0 4.8%
H...C 13.5%
H...H 35.5%
H...N 5.5%
d_e
d_i
(Å)

（a）

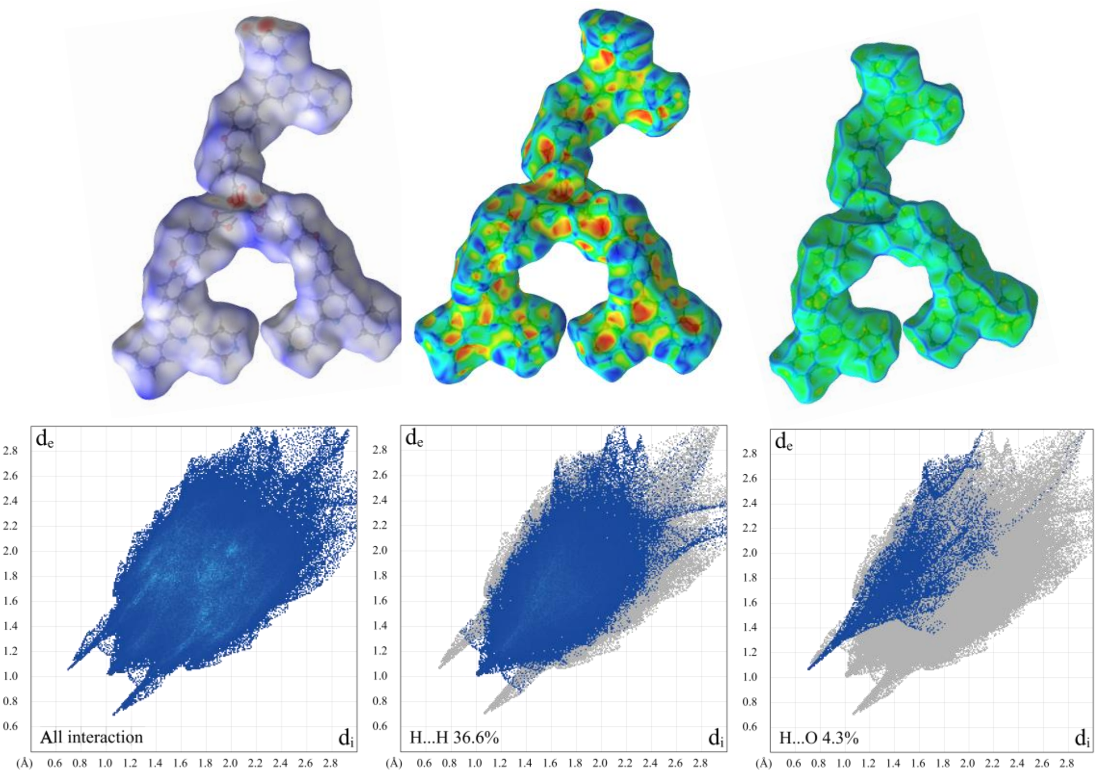
All interaction
H...H 36.6%
H...O 4.3%
d_e
d_i
(Å)

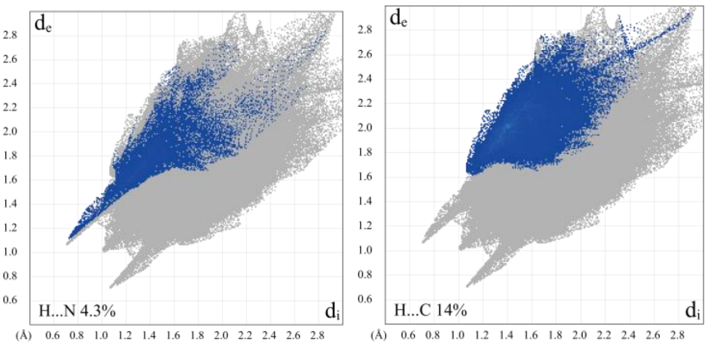
d_e
d_i
H...N 4.3%
H...C 14%
(Å)

（b）

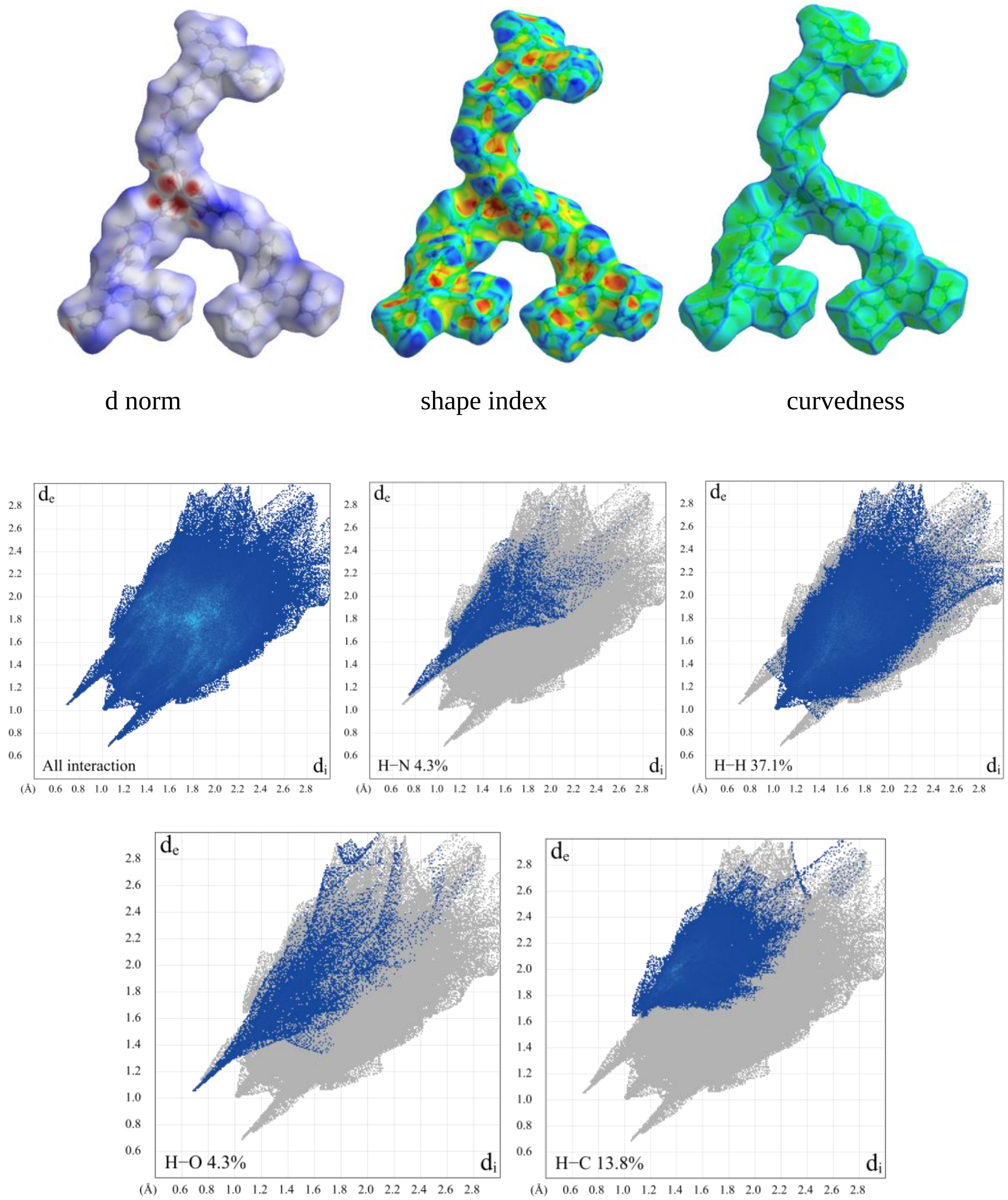
d norm
shape index
curvedness
d_e
d_i
All interaction
H−N 4.3%
H−H 37.1%
H−O 4.3%
H−C 13.8%
(Å)

（c）

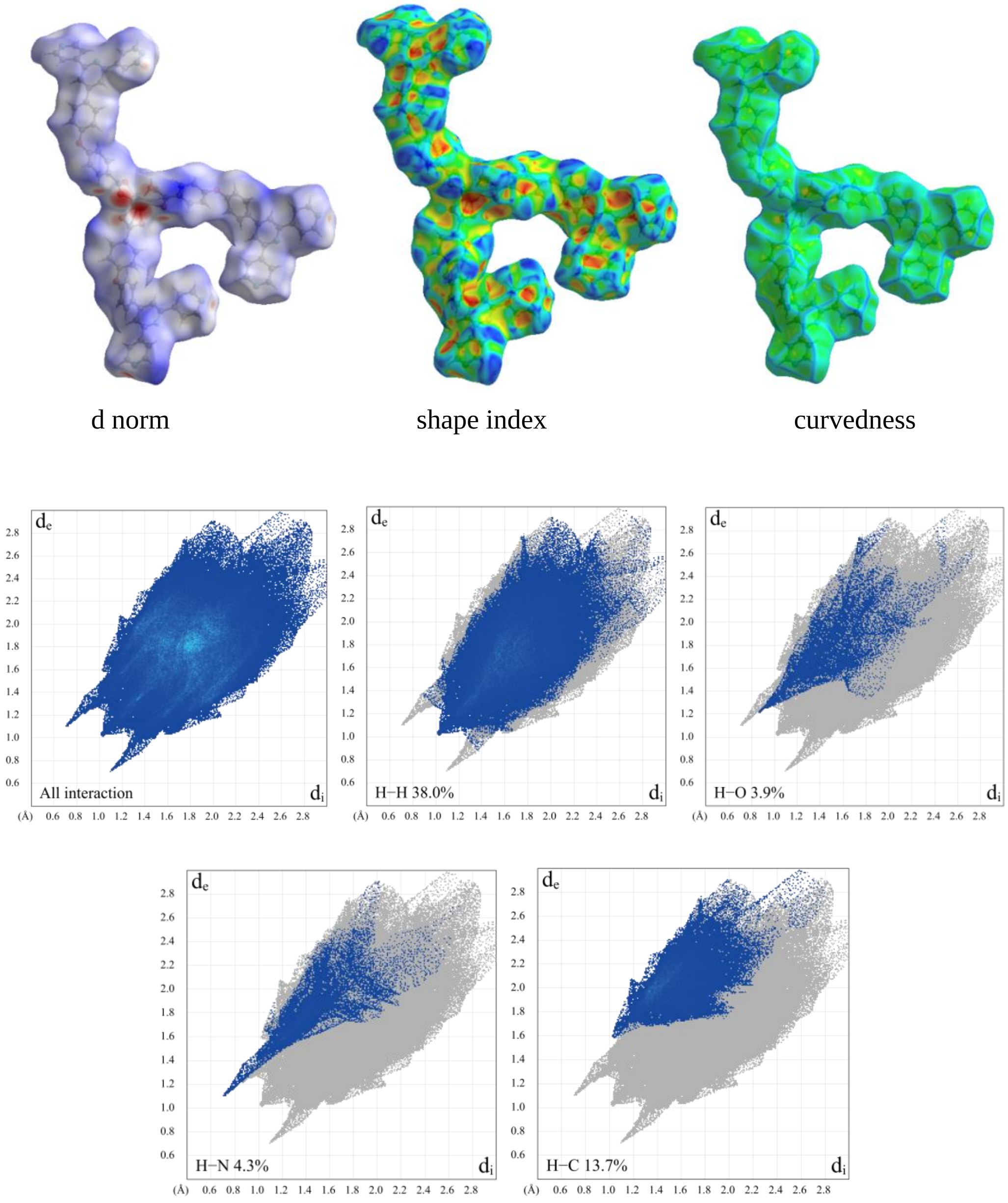
d norm
shape index
curvedness
d_e
d_i
All interaction
H–H 38.0%
H–O 3.9%
H–N 4.3%
H–C 13.7%
(Å)
0.6 0.8 1.0 1.2 1.4 1.6 1.8 2.0 2.2 2.4 2.6 2.8

（d）

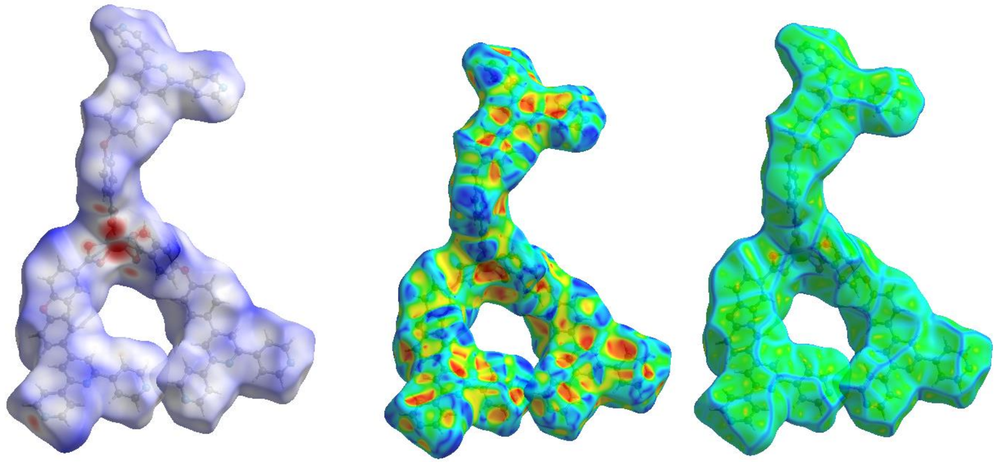

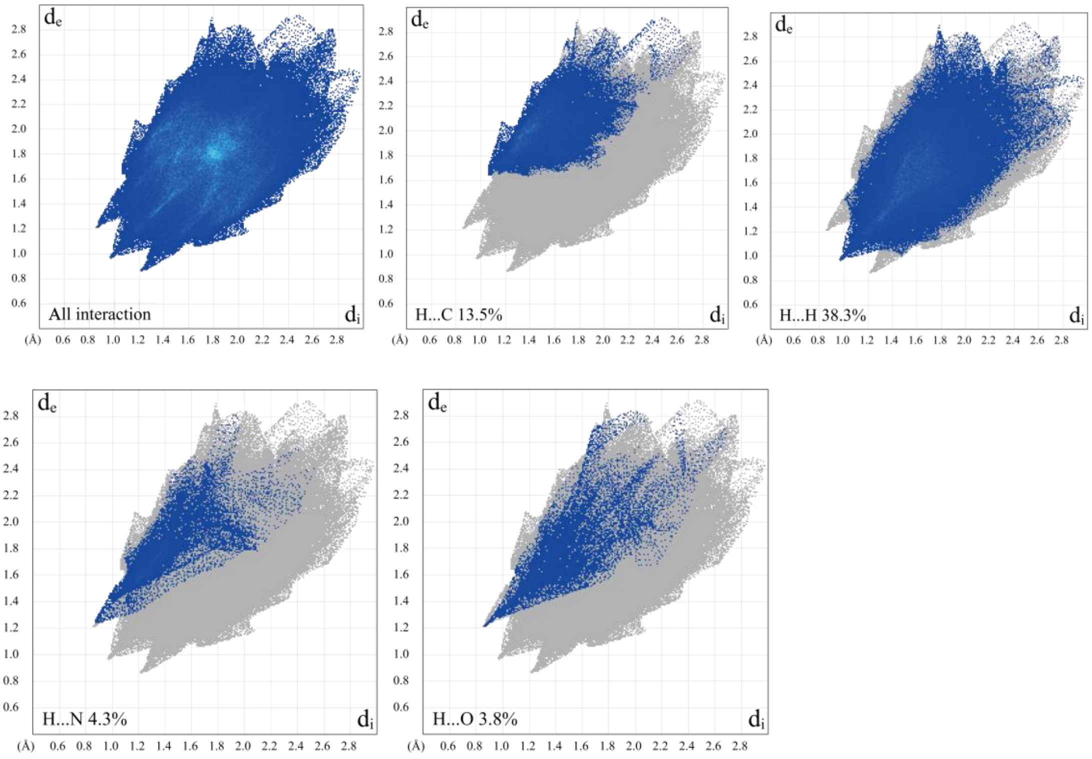
All interaction
H...C 13.5%
H...H 38.3%
H...N 4.3%
H...O 3.8%
d_e
d_i
(Å)

（e）

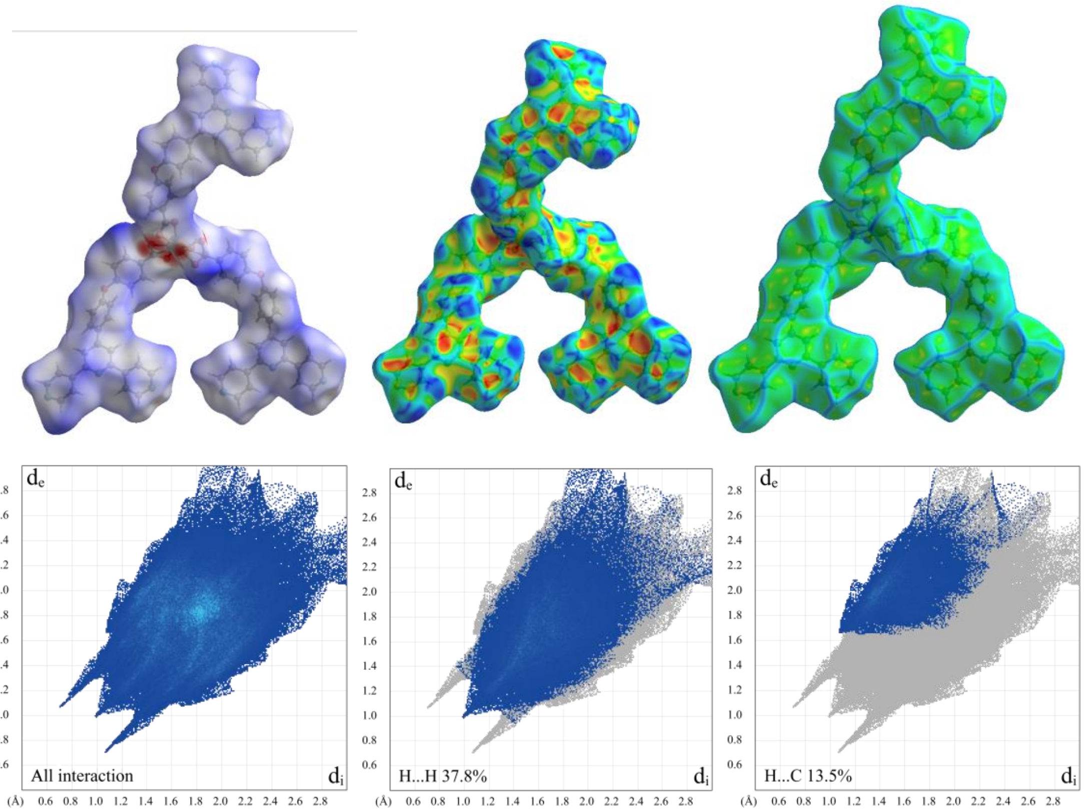
All interaction
H...H 37.8%
H...C 13.5%
d_e
d_i
(Å)

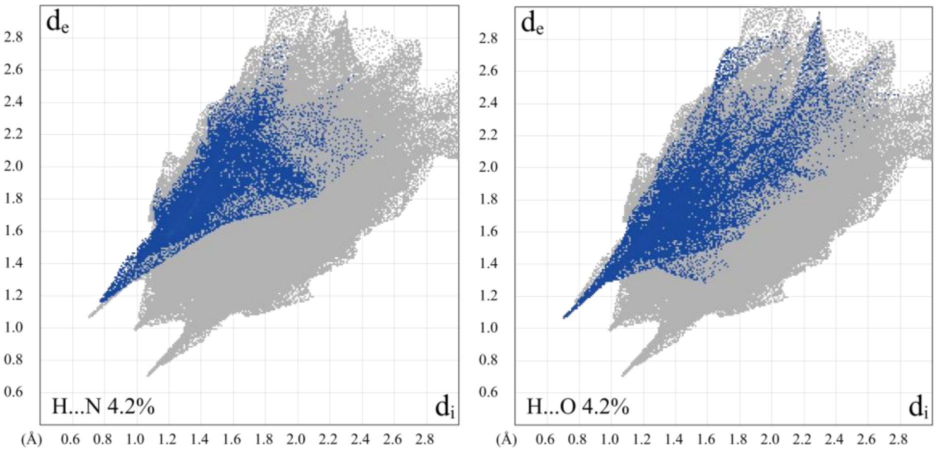

（f）

FIGURE S5 Hirshfeld surface mapped with dnorm (left), shape index (middle), and curvedness (right) for 1 (a), 2 (b), 3 (c),5 (d), 6 (e) and 7 (f)

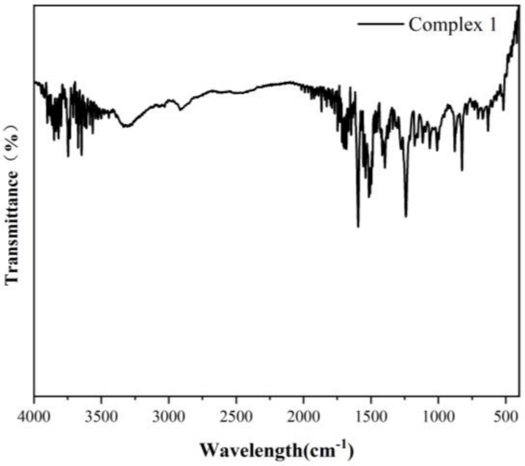

(a)

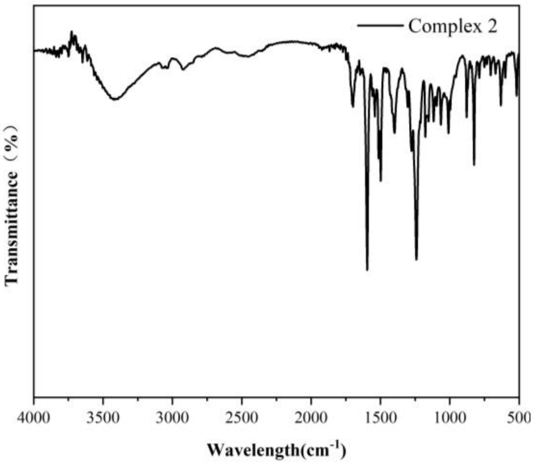

(b)

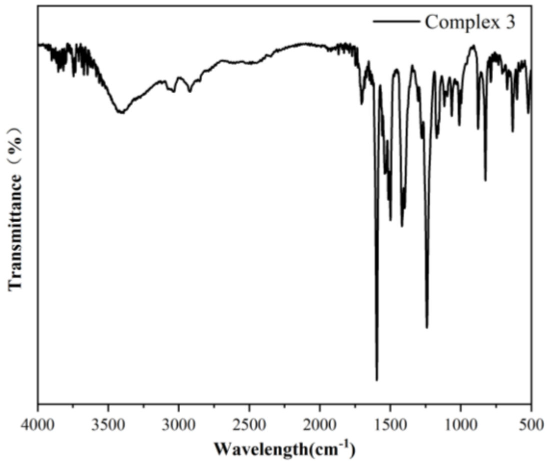

(c)

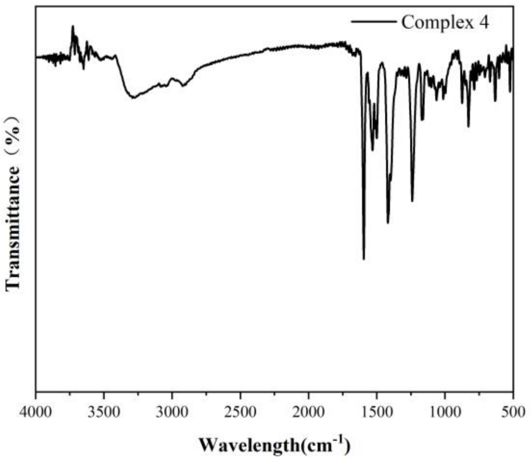

(d)

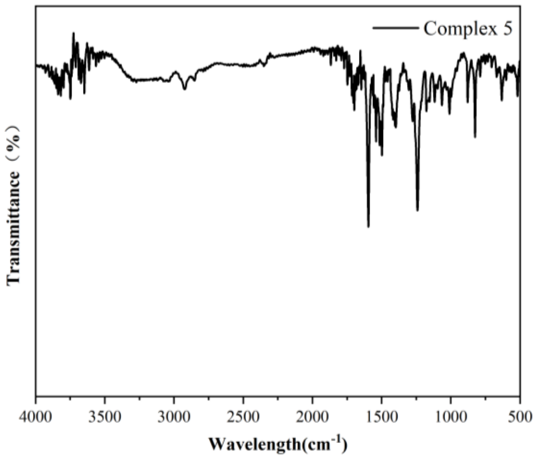

(e)

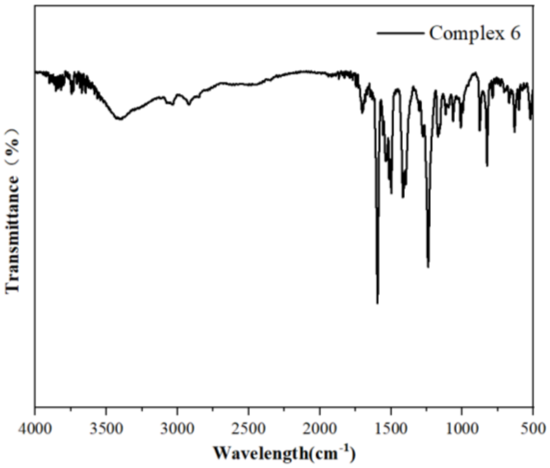

(f)

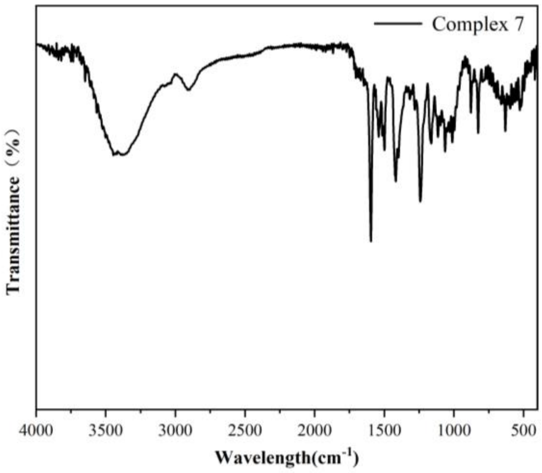

(g)

FIGURE S6 The IR spectra for complex1(a), 2 (b), 3 (c),4 (d),5(e), 6(f) and 7(g)
